# MATEO: intermolecular α-amidoalkylation theoretical enantioselectivity optimization. Online tool for selection and design of chiral catalysts and products

**DOI:** 10.1186/s13321-024-00802-7

**Published:** 2024-01-23

**Authors:** Paula Carracedo-Reboredo, Eider Aranzamendi, Shan He, Sonia Arrasate, Cristian R. Munteanu, Carlos Fernandez-Lozano, Nuria Sotomayor, Esther Lete, Humberto González-Díaz

**Affiliations:** 1 Department of Organic and Inorganic Chemistry, Faculty of Science and Technology, University of The Basque Country (UPV/EHU), P.O. Box 644, 48080 Bilbao, Spain; 2 Department of Computer Science and Information Technologies, Faculty of Computer Science, CITIC-Research Center of Information and Communication Technologies, University of A Coruña, Campus Elviña s/n, 15071 A Coruña, Spain; 3 IKERDATA S.L., ZITEK, University of Basque Country UPVEHU, Rectorate Building, 48940 Leioa, Spain; 4 IKERBASQUE, Basque Foundation for Science, 48011 Bilbao, Spain

**Keywords:** Chiral phosphoric acid catalysts, Cheminformatics, Machine learning, Amidoalkylation

## Abstract

The enantioselective Brønsted acid-catalyzed α-amidoalkylation reaction is a useful procedure is for the production of new drugs and natural products. In this context, Chiral Phosphoric Acid (CPA) catalysts are versatile catalysts for this type of reactions. The selection and design of new CPA catalysts for different enantioselective reactions has a dual interest because new CPA catalysts (tools) and chiral drugs or materials (products) can be obtained. However, this process is difficult and time consuming if approached from an experimental trial and error perspective. In this work, an Heuristic Perturbation-Theory and Machine Learning (HPTML) algorithm was used to seek a predictive model for CPA catalysts performance in terms of enantioselectivity in α-amidoalkylation reactions with R^2^ = 0.96 overall for training and validation series. It involved a Monte Carlo sampling of > 100,000 pairs of query and reference reactions. In addition, the computational and experimental investigation of a new set of intermolecular α-amidoalkylation reactions using BINOL-derived *N*-triflylphosphoramides as CPA catalysts is reported as a case of study. The model was implemented in a web server called MATEO: InterMolecular Amidoalkylation Theoretical Enantioselectivity Optimization, available online at: https://cptmltool.rnasa-imedir.com/CPTMLTools-Web/mateo. This new user-friendly online computational tool would enable sustainable optimization of reaction conditions that could lead to the design of new CPA catalysts along with new organic synthesis products.

**Supplementary Information:**

The online version contains supplementary material available at 10.1186/s13321-024-00802-7.

## Introduction

Chiral Phosphoric Acid (CPA) and related catalysts are widely recognized and versatile tools in catalysis and organic synthesis useful for the synthesis of chiral drugs products [1–3]. The selection and design of new CPA catalysts for different enantioselective reactions has a dual interest because new CPA catalysts (tools) and chiral drugs or materials (products) can be obtained [4]. However, this process is difficult and time consuming if approached from an experimental trial and error perspective. Quantum Computational Chemistry tools may help to unravel the mechanism of reactions and help in the design of new CPA catalysts [5, 6]. Unfortunately, these techniques are less useful when it is necessary a fast scanning/optimization of new CPA catalysts for large libraries of reactions with diverse substrates, nucleophiles, products, and conditions (temperature, time, catalyst load, etc.). Cheminformatics methods relying upon Artificial Intelligence/Machine Learning (AI/ML) algorithms could help to speed up the discovery of new molecules [7–9] and in the design new chiral catalysts and products without engaging in a long term, empirical or quantum investigation [10–13]. Therefore, there is a need to develop fast-track computational tools able to predict the enantiomeric excess saving time and experimental resources. However, the application of AI/ML techniques to the study of enantioselective reactions is still uncommon due to the inherent complexity of the problem. In addition, most models are not implemented in public online web servers or they are not available for researchers or companies. In this context, it is remarkable Sigman’s et al. platform for CPA catalysts and organophosphorous ligand design [14, 15]. In these works, the authors predict reactivity using structural information of the query reactants/products. However, useful experimental/operational conditions of already known reference reactions similar to the query reaction are not considered. Recently, our group has faced this problem by introducing the Perturbation-Theory and Machine Learning (PTML) approach that employs as inputs both vectors of structural variables **D**_kqi_ and vectors of multiple experimental conditions **c**_qj_. These PTML algorithms have been applied in medicinal chemistry, vaccine design, nanotechnology, and in catalysis as well [16–21]. In fact, we have previously reported a preliminary PTML model for the design of CPA catalysts for intermolecular α-amidoalkylation reactions [22]. However, the model was not implemented on a public online web server and is difficult to use by an experimentalist.

**Scheme 1:**
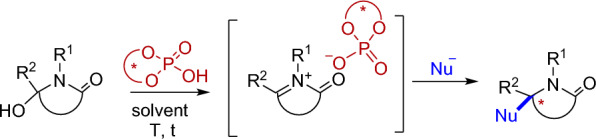
General scheme for CPA-catalyzed intermolecular α-amidoalkylation reactions

Consequently, in this work, we are going to focus on the development of a public web server for the selection and design of CPAs catalysts for enantioselective intermolecular α-amidoalkylation reactions (Scheme 1). In these reactions, the protonation of an α-hydroxylactam by the CPA would give a chiral conjugate base/*N*-acyliminium ion pair, which would be trapped by a nucleophile enantioselectively, generating a new tertiary or quaternary stereocenter [23, 24]. The α-amidoalkylation reaction of aromatic systems using *N*-acyliminium ions as electrophiles is a Friedel–Crafts-type reaction that has found widespread application in organic synthesis for the production of new drugs and natural products [25, 26]. For example, we have applied the procedure to the enantioselective synthesis of Nuevamine type alkaloids. Thus, indol and acyl moieties can be easily introduced in the alpha position of the nitrogen atom, using sterically demanding BINOL-derived CPA catalyst [27]. However, the enantioselectivity of these CPA catalyzed reactions is sensitive to many factors, from the nature of the nucleophile and the catalyst to the experimental conditions (solvent, temperature, etc.). In this context, many efforts have been made to understand the role of non-covalent interactions in organocatalyzed reactions and to rationalize and predict their stereochemical outcome using Quantum Chemical methods [28–30]. However, the chemical space accessible by organic synthesis is very wide, and all compatible combinations of substrate, nucleophile, catalyst, and solvent should have to be scanned.

Therefore, the use of Cheminformatics models to explore the chemical space of these reactions becomes a very interesting option in order to reduce costs and time. Therefore, we decided to develop a new user-friendly online computational tool able to carry out screenings of this CPA-catalyzed intermolecular α-amidoalkylation reaction space for a large number of chiral catalysts, substrates, nucleophiles, solvents, chiral products, and reaction conditions. First, we carried out a re-evaluation of all the available data in our record to obtain a better estimate of the chemical space of these reactions. Next, we developed a new PTML model using Heuristics and Monte Carlo sampling calculations without relying on costly computational calculations. This PTML model was able to predict the enantioselectivity with R^2^ = 0.96 after a comparative study 332 reactions, which can be paired in > 100,000 ways, as each reaction can be a query or reference reaction.

**Scheme 2:**
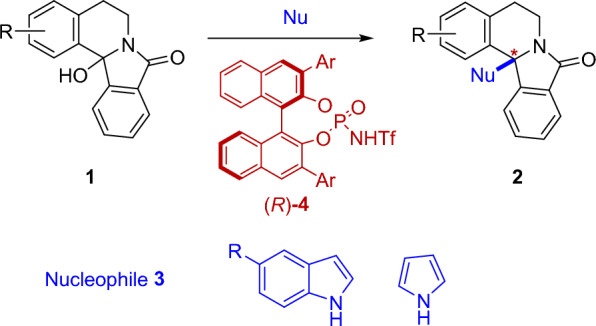
Chiral BINOL-derived *N*-triflylphosphoramide-catalyzed intermolecular α-amidoalkylation reactions

Later, we developed the web server called MATEO (interMolecular Amidoalkylation Theoretical Enantioselectivity Optimization), which is available at the online platform CPTMLTool (https://cptmltool.rnasa-imedir.com/). Finally, we have illustrated the practical use of the online tool with the experimental-theoretical study of a new set of CPA-catalyzed α-amidoalkylation reactions starting from bicyclic α-hydroxylactams **1** to construct the isoindoloisoquinoline framework **2** with a quaternary stereocenter. Electron-rich heteroaromatics (indole and pyrrole derivatives) **3** will be used as nucleophiles and chiral BINOL-derived *N*-triflylphosphoramides **4** as catalysts (Scheme 2). This new tool may help experimentalists in organic, medicinal, and materials chemistry to explore the chemical space of CPA-catalyzed α-amidoalkylation reactions and to optimize the reaction conditions for practical purposes.

## Materials and methods

### Dataset and parameter studied

In this paper, we have carried out the study of the enantiomeric excess *ee*_*R*_(%)_obs_ parameter in intermolecular α-amidoalkylation reactions. The value *ee*_*R*_(%)_obs_ allows to quantify the enantiomeric excess by applying an (*R*)-catalyst. This parameter is represented as *ee*_*R*_(%)_obs_ = Sign(Prod)·Sign(Cat*R*)·*ee*(%)_obs_, where Sign(Prod) = 1 for (*R*)-product or Sign(Prod) = − 1 for (*S*)-product, taking into account an *R* or *S* notation of products experimentally obtained consistent with the Cahn-Ingold-Prelog (CIP) rules [31]. The function Sign(Cat) = 1 for all reactions carried out with an (*R*)-catalyst, irrespective of the product obtained. On the other hand, the sign was switched from + 1 to Sign(Cat) = − 1 for the reactions carried out with (*S*)-catalyst and the sign Sign(Prod) was changed to the opposed. This operation transform (*S*)-catalyst reactions into (*R*)-catalyst reactions with the same absolute value of enantiomeric excess but opposed sign of *ee*_*R*_(%)_obs_. All reactions are expected to give the same result but with inverse configuration when you change the chirality of the Catalyst. Consequently, all reactions were studied as if they have been performed using an (*R*)-catalyst keeping the (*R*)-catalyst when originally used or switching the signs of Sign(Prod) and Sign(Cat) for (*S*)-catalyst reactions. In practice, this procedure will allow us to omit the use of chiral molecular descriptors for substrates, products, catalysts, etc., because all the chirality information will be included in the *ee*_*R*_(%) terms for the query or reference reactions (see next sections). In fact, the method worked properly in this specific case because all the reactions give products with only one stereogenic center. Consequently, we have all the chirality information necessary included in both sides of the equation without necessity of using chiral molecular descriptors.

### Reaction condition variables

Apart from defining the molecular descriptors, we also consider different reaction conditions variables V_k_(c_qi_) as input variables in order to quantify a k^th^ property (k = 1, 2, 3) related to a general reaction condition (c_q_) and/or specific reactant. In this chemical reaction dataset, the variables taken into account for the i^th^ query reactions were: V_1_(c_qi_) = T(^o^C) = Temperature, V_2_(c_qi_) = t(h) = reaction time and V_3_(c_qi_) = L(%) = catalyst loading. By analogy, the values of variables considered for each j^th^ reference reactions were: V_1_(c_rj_) = T(^o^C) = Temperature, V_2_(c_rj_) = t(h) = reaction time, and V_3_(c_rj_) = L(%) = catalyst loading.

### Dataset studied, compounds and reactions notation

A dataset of 332 CPA-catalyzed enantioselective intermolecular α-amidoalkylation reactions has been compiled, which comprised 324 reactions obtained from literature (see Additional file 3) and 8 new reactions studied in this work for the first time (see Table 8). These reactions have been grouped into 34 families according to the different structural patterns of the substrates, nucleophiles, and catalysts. There are different types of substrates **S** (mostly cyclic and bicyclic α-hydroxylactams, but also 3-hydroxyindolines) that are reacted with different types of nucleophiles **Nu** (indoles, pyrroles, Hantzsch esters, enols and enamides) using CPAs (phosphoric acids or the corresponding *N*-triflylphosphoramides and sulfonamides) as catalysts **Cat**.

**Scheme 3.**
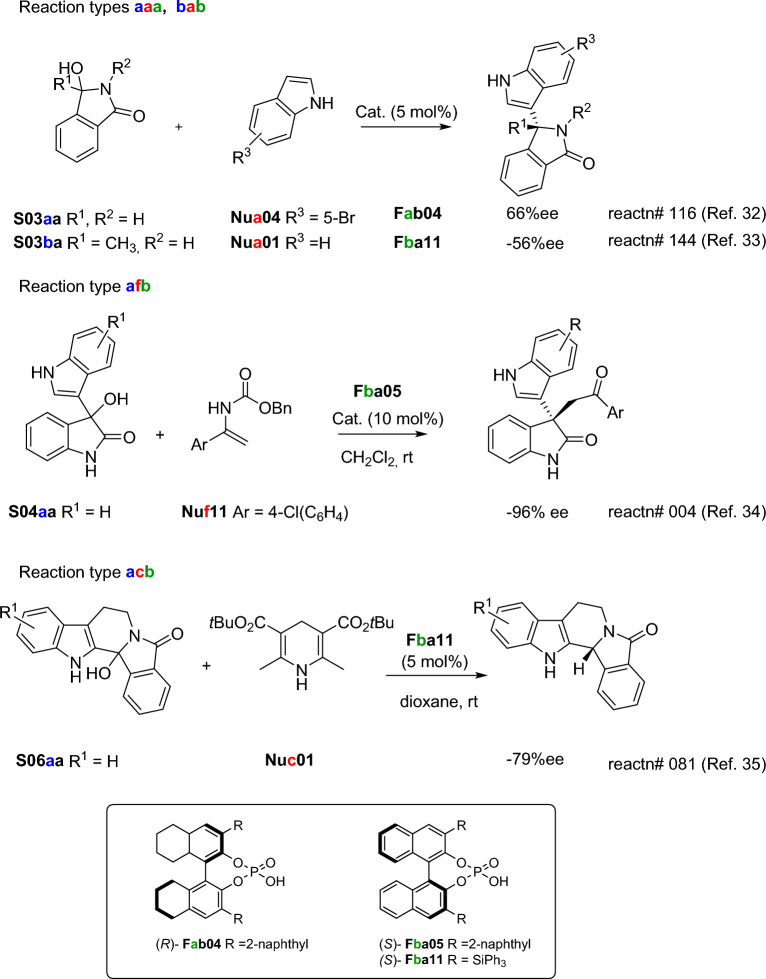
Selected examples of intermolecular α-amidoalkylation reactions included in the reference dataset, including molecule coding and reaction number (for Additional file 3)

All compounds have been labeled with a 5-element code Xyznn, X = S for Substrates, X = Nu for Nucleophiles, and X = Family of Catalysts; y = is the structural family (a, b, c,…), z = is the structural sub-family, if any (a, b, c, …), and nn = is the ID number of the compound in the dataset. When the structural sub-family is missing, the label y in the notation is omitted. Then, a code was created to classify each reaction in the dataset into different reactions types based on the structure of the molecules involved. Thus, the values of the family label y of the Substrate, Nucleophile, and Catalyst were concatenated in this order to obtain the ID code of each reaction type. For example, the reaction of the Substrate **S03aa** with the Nucleophile **Nua04** and the Catalyst **Fab04** belongs to the reaction type with the ID code **aaa.** Scheme 3 shows selected examples of different reaction types included in the dataset using different types of cyclic hydroxylactams as substrates (**S03**, **S04**, **S06**) and different nucleophiles, such indoles (**Nua**) [32, 33] enamides (**Nuf**) [34] or Hantzsch esters as reducing agents (**Nuc**) [35], with CPAs catalysts (**F**). The full experimental detail of each of the 324 reference reactions (substrate, nucleophile, catalysts, catalyst loading product, solvent, temperature, time, yield, % ee) is included in the Supporting Information (Additional file 3), which also includes the SMILE code of the substrate, nucleophile and catalyst in each case. To have a general view of the chemical space in the dataset, general schemes for all reactions included in the reference dataset are included in the Supporting Information (Additional file 1: Schemes S1 to S9). The structures and codification of substrates (**S**), nucleophiles (**Nu**), and catalysts (**cat.**) is included in the Supporting Information (Additional file 1).

### Molecular descriptors calculation

First, the web tool MMDcalc was used to calculate the molecular descriptors D_k_(m_sqi_)_g_ and D_k_(m_sri_)_g_ of the molecules m_sqi_ and m_sri_ involved in the query and reference reactions [36]. The MMDcalc tool is an online web server available at the PTMLTool platform (https://cptmltool.rnasa-imedir.com/) for public use. This tool implements the Markov Chain Invariants for Networks Simulation and Design (MARCH-INSIDE) algorithm online. MARCH algorithm uses Markov Chains to calculate the average value of different atomic properties. These average values of atomic properties are calculated for predefined groups of atoms (g) inside the molecule and all their neighbors placed at topological distance (d). In the notation D_k_(m_sqi_)_g_/D_k_(m_sri_)_g_ the letter D = Descriptor, k = type of descriptor, s = sub-type of molecule, q = molecules involved in query reaction, r = molecules involved in reference reaction, i = ID number of the molecule, g = group of atoms inside the molecule. The general formula for the calculation is shown in Eq. 1 (see MARCH-INSIDE algorithm details in literature) [37].

1 $${{D}_{k}({m}_{sqi})}_{g}=\frac{1}{{d}_{max}}\sum_{d=1}^{{d}_{max}}\sum_{a\in g}^{\forall a\in g}{{D}_{d}({m}_{sqi})}_{a}= \langle {{D}_{d}({m}_{sqi})}_{a}\rangle$$

The k^th^ types (k = 1, 2, 3, 4, and 5) of molecular descriptors are: D_1_ = Number of Valence Electrons (Zv), D_2_ = van der Waals Volume (Vvdw), D_3_ = Sanderson Electronegativity (χ), D_4_ = Polarizability (α), and D_5_ = Electron Affinity (EA). The sub-types (s) of query molecules m_sqi_(s = 1, 2, 3, 4, and 5) are: m_1qi_ = Substrate_qi_, m_2qi_ = Nucleofile_qi_, m_3qi_ = Catalyst_qi_, m_4qi_ = Solvent_qi_, and m_5qi_ = Product_qi_. The chemical functional groups or atom groups G_g_ (g = 1, 2, 3, 4, 5) are the following: G_1_ = Saturated Carbon atoms (C_sat_), G_2_ = Unsaturated Carbon atoms (C_uns_), G_3_ = Heteroatoms (Het), G_4_ = NonHalogen (X) Heteroatoms (HetNoX), and G_5_ = Total (Tot). The groups of atoms indicate which atoms in the molecules were used as the basis for calculating the different local (g < 5) and/or total (g = 5) molecular descriptors.

### ML linear model

In this section, D_k_(m_sqi_)_g_ values were introduced in order to look for a linear ML model. It is worth mentioning that each entry line of the dataset denotes only one query reaction (R_qi_). The enantiomeric excess *ee*_R_(%)_qicalc_ of the query reaction (R_qi_) was predicted by applying both variables V_k_(c_qi_) as input depending on the experimental conditions and the molecular descriptors D_k_(m_sqi_)_g_ of the molecules taken into consideration in the reaction. With both sets of variables as inputs, we can seek a linear AI/ML additive model. A best practice, the following equality holds *ee*_R_(%)_calcqi_≈ *ee*_R_(%)_qiobs,_ when the additive linear hypothesis is correct. The general additive form of AI/ML model to be developed is the following.

2 $${{ee}_{R}\left(\%\right)}_{calcqi}=\sum_{k=1}^{{k}_{max}}\sum_{s=1}^{{s}_{max}}{a}_{k,s}\cdot {V}_{k}({c}_{qi})+ \sum_{k=1}^{{k}_{max}}\sum_{s=1}^{{s}_{max}}\sum_{g=1}^{{g}_{max}}{b}_{k,s,g}\cdot {{D}_{k}({m}_{sqi})}_{g}+{e}_{0}$$

### PTML linear model

The PTML model is a well-known approach that can be used to predict the reactivity of a new case (reaction) through making comparisons with other known reactions. Our model can provide as output the *ee*_*R*_(%)_calcqi_. On the other hand, the *ee*_*R*_(%)_calcqi_ is calculated for a query reaction(R_qi_) due to the observed enantiomeric excess *ee*_*R*_(%)_rjobs_ = *ee*_*R*_(%)_refj_ of a reaction (R_rj_) used as reaction of reference is already known. For this reason, the dataset applied to train/validate the PTML model, each entry line takes into consideration a pair of reactions, specifically a query reaction compared to a reference reaction (R_qi_ *vs*. R_rj_). The PTML linear model enables to predict *ee*_*R*_(%)_calci_ starting with the experimental value of *ee*_*R*_(%)_refj_ of a reference reaction. Afterwards, the model includes the influences of different structural, operational or experimental conditions variations (perturbations) in the query in regard to the reference reaction. We use PT Operators (PTOs) in order to quantify these variations or perturbations. The parameter of PTOs are denoted as the form ΔD_k_(m_sqi_, m_srj_)_g_ for structural variations and ΔV_k_(c_qi_, c_rj_) for variations in the experimental reactions conditions. The formula of the PTML models used in this section are shown in Eqs. 3 and 4;

3 $${{ee}_{R}\left(\%\right)}_{calcqi}={{ee}_{R}\left(\%\right)}_{refj}+\sum_{k=1}^{{k}_{max}}\sum_{i=1}^{{i}_{max}}{a}_{k,i}\cdot {\Delta V}_{k}({c}_{qi},{c}_{rj})+\sum_{k=1}^{{k}_{max}}\sum_{s=1}^{{s}_{max}}\sum_{g=1}^{{g}_{max}}{b}_{k}\cdot {{\Delta D}_{k}({m}_{sqi},{m}_{srj})}_{g}+{e}_{0}$$

4 $${{ee}_{R}\left(\mathrm{\%}\right)}_{calcqi}={{ee}_{R}\left(\mathrm{\%}\right)}_{refj}+\sum_{k=1}^{{k}_{max}}\sum_{i=1}^{{i}_{max}}{a}_{k,i}\cdot \left[{V}_{k}\left({c}_{qi}\right)-{V}_{k}\left({c}_{rj}\right)\right]+\sum_{k=1}^{{k}_{max}}\sum_{i=1}^{{i}_{max}}\sum_{g=1}^{{g}_{max}}{b}_{k}\cdot \left[{{D}_{k}({m}_{sqi})}_{g}-{{D}_{k}({m}_{srj})}_{g}\right]+{e}_{0}$$

In this work, the linear additive model used as a function of reference *ee*_R_(%)_robs_ and two sets of PTOs represented by ΔV(c_qi_, c_rj_) and ΔD(m_sqi_, m_srj_)_g_ as input. The function of reference *ee*_R_(%)_robs_ is equal to the observed values of enantiomeric excess *ee*(%), when the reference reaction used a (*R*)-catalyst with *R* configuration. We have developed two types of PTO in order to seek the PTML linear model. On the one hand, the first type of PTO is described as ΔV_k_(c_qi_, c_rj_) = [V_k_(c_qi_)–V_k_(c_rj_)]. It takes into account the perturbations/deviations in the values of the k^th^ variables/conditions of reactions V(c_qi_) of the q^th^ query reaction against the original values of the same variables V_k_(c_r_) for the r^th^ reaction of reference. On the other hand, the second type of PTO is denoted as: ΔD_k_(m_sqi_, m_srj_) = [D_k_(m_sqi_) – D_k_(m_srj_)]_g_. It considers the perturbations/deviations in the values of the molecular descriptors of the query with respect to the reference molecules. Subsequently, the input variables for the reaction of the reference V_k_(c_rj_) are related to a k^th^ property (k = 1, 2, 3). The connection between the input variables and k^th^ property enables the connection in terms of general experimental conditions of reaction (c_rj_) and/or specific reactants: V_1_(c_rj_) = T(^o^C) = Temperature, V_2_(c_rj_) = t(h) = reaction time, and V_3_(c_rj_) = L(%) = catalyst loading, for the reaction of reference (R_rj_). The input variables denoted as D_k_(m_ri_)_g_ are the molecular descriptors of type k^th^ for the i^th^ molecules (m_sri_) of type q^th^ involved in the reference reaction (R_rj_). Analogously, the molecules m_ri_ taken part in the reaction of reference are m_r1j_ = Substrate_rj_, m_r2j_ = Nucleofile_j_, m_r3j_ = Catalyst_rj_, and m_4rj_ = Solvent_rj_. In addition, we use the k^th^ types of molecular descriptors as the same way as for the query reaction D_1_ = Number of Valence Electrons (Zv), D_2_ = Van der Waals Volume (Vvdw), D_3_ = Sanderson Electronegativity (χ), D_4_ = Polarizability (α), and D_5_ = Electron Affinity (EA). In Table 1, we illustrate the detailed information about of all the PTOs used as input variables in the PTML models.

**Table 1:** Definition of variables used as inputs of the PTML model

Experimental conditions (c_q_)	Perturbation operators^b^	Type of operator
Reaction temperature (T)	ΔV(T) = ΔT = T_q_–T_r_	Temperature deviation
Reaction time (t)	ΔV(t) = Δt = t_q_–t_r_	Time deviation
Catalyst loading [Load (%)]	ΔV(Load(%)) = Load(%)_q_–Load(%)_r_	Conc. difference
Molecules (m_q_)^a^	Perturbation terms	Type of operator^a^
Substrate (Sub)	ΔD_k_(Sub_qi_, Sub_rj_)_g_ = [D_k_(Sub_qi_)_g_–D_k_(Sub_rj_)]_g_	Structural variation
Product (Prod)	ΔD_k_(Prod_qi_, Prod_rj_)_g_ = [D_k_(Prod_qi_)_g_–D_k_(Prod_rj_)]_g_	
Nucleophile (Nuc)	ΔD_k_(Nuc_qi_, Nuc_rj_)_g_ = [D_k_(Nuc_qi_)_g_–D_k_(Nuc_rj_)]_g_	
Catalyst (Cat)	ΔD_k_(Cat_qi_, Cat_rj_)_g_ = [D_k_(Cat_qi_)_g_–D_k_(Cat_rj_)]_g_	
Solvent (Solv)	ΔD_k_(Solv_qi_, Solv_rj_)_g_ = [D_k_(Solv_qi_)_g_–D_k_(Solv_rj_)]_g_	

^a^Molecules (m) involved in the reaction with distinguishable roles: m_qsi_ = Substrate (Sub_q_), Product (Prod_q_), Nucleophile (Nuc_q_), Catalyst (Cat_q_), and Solvent (Solv_q_)

^b^PTOs with formula ΔV(m_q_, m_r_)_g_ = [V(m_q_)_g_–V(m_r_)]_g_. These PTOs measure the variation of the value of the molecular property/structural variable (V) in the query molecules m_q_with respect to the value for molecule m_r_ with the same role in the reaction of reference. The values of V_k_(m_q_)_g_ are average values of the properties V_k_ = Sanderson Electronegativities (χ), Polarizabilities, etc., for all the atoms in the group g and all their neighboring atoms placed at a topological distance k ≤ 5. Consequently, these properties have been calculated for all the atoms in the molecule (Tot) or for subsets of atoms (group g). The groups of atoms studied are g = unsaturated carbons (C_uns_), saturated carbons (C_sat_), Heteroatoms (Het), Heteroatoms non-Halogen (HetNoX)

### AI/ML *vs.* PTML linear model development

So as to seek the AI/ML and PTML linear models, we apply Multivariate Linear Regression (MLR) and Linear Neural Network (LNN) algorithms by using the software STATISTICA [38]. In this sense, in the PTML regression models, the values of observed (experimental) enantiomeric excess *ee*_R_(%)_obsqi_ against multiple values of reference *ee*_R_(%)_refj_ have to be fitted. The regression model allows to generate artifacts in the standard distribution of the data [39]. The parameters a_k,s_ b_k,s,g_ and e_0_ are the coefficients of the model to be fitted by AI/ML algorithms. The formula for the PTML linear regression models was fitted as presented in the Eq. 5;

5 $$\Delta {{ee}_{R}\left(\%\right)}_{qi}=\sum_{k=1}^{{k}_{max}}\sum_{s=1}^{{s}_{max}}{a}_{k,s}\cdot {\Delta V}_{k}({c}_{qi},{c}_{rj})+\sum_{k=1}^{{k}_{max}}\sum_{s=1}^{{s}_{max}}\sum_{g=1}^{{g}_{max}}{b}_{k,s,g}\cdot {{\Delta D}_{k}({m}_{sqi},{m}_{srj})}_{g}+{e}_{0}$$

### HPTML linear model

The PTML linear model built can predict diverse outputs for the same reaction taking into consideration the selected reference reactions. Therefore, in this section we introduced different Heuristics (H) in order to define the best reaction performance or set of reactions as reference. In this work, specifically we used two following heuristic. On the one hand, the first heuristic (H_1_) can calculate the final predicted value as this form: *ee*_R_(%)_qrpred_ = *ee*_R_(%)_qrmin_. This value is obtained using as reference the reaction with a minimum (Min) value of the PTOs in other words, the minimal deviation. Specifically, the heuristic (H_1_) uses as reference, the reaction with a minimal difference/deviation (Δ) between the input variables ΔV(m_qsi_, m_rsj_) and ΔV(c_qi_, c_rj_) for all (∀) pairs of reactions. On the other hand, the second heuristic (H_2_) can calculate the value *ee*_R_(%)_qrpred_ = *ee*_R_(%)_qravg_ = Avg(*ee*_R_(%)_qrcalc_). Particularly, the heuristic (H_2_) uses as reference the values of variables ΔD(m_qi_, m_rj_) (molecule structural variations) and ΔV(c_qi_, c_rj_) (experimental conditions variations) for all (∀) pairs of reactions. As the first step, we calculated the 331 different *ee*_R_(%)_qrcal_ values, not including the query. Then, we obtained the final values as the average for all the references. These two heuristics can be described as illustrated in Eqs. 6 and 7.

6 $${H}_{1}:{ ee}_{R}{\left(\mathrm{\%}\right)}_{{\text{qrpred}}}={ ee}_{R}{\left(\mathrm{\%}\right)}_{{\text{qrmin}}}\stackrel{\forall q,r}{\Rightarrow }Min\left\{PTOs\left[\mathrm{\Delta V}\left({{\text{m}}}_{qi},{{\text{m}}}_{rj}\right),\mathrm{ \Delta V}\left({{\text{c}}}_{qi}, {{\text{c}}}_{rj}\right)\right]\right\}$$

7 $${H}_{2}:{ ee}_{R}{\left(\mathrm{\%}\right)}_{{\text{qrpred}}}={ ee}_{R}{\left(\mathrm{\%}\right)}_{{\text{qravg}}}\stackrel{\forall q,r}{\Rightarrow }Avg\left\{PTOs[(\mathrm{\Delta V}\left({{\text{m}}}_{qi},{{\text{m}}}_{rj}\right),\mathrm{ \Delta V}\left({{\text{c}}}_{qi}, {{\text{c}}}_{rj}\right)]\right\}$$

### Monte carlo simulation

Most reactivity prediction models already reported take into consideration only the structure of the reactants but omit the values of temperature, catalyst loading, time of reaction, solvent polarity, etc. when predicting the enantiomeric excess of the reactions. In fact, many of the works focus only on yield at specific conditions of T, time, load, etc., and do not predict the enantiomeric excess. In addition, the values of enantiomeric excess, T, time, load, solvent polarity, etc. when measured experimentally contains a certain degree of error because most researchers do not measured them for triplicate or lead them uncontrolled like when using room temperature conditions. In this context, the Monte Carlo Simulation (MC) starts with the original values of the non-structural variables T, t, Load and using a random generator creates new values with small variations with respect to the original values. MC experiments are a wide-ranging class of computational algorithms that base on repeated random sampling to obtain numerical results. This method are among the most useful data sampling in Cheminformatics [40–42].

In this work, we used an MC algorithm to predict the enantiomeric excess of the reactions taking into consideration all these factors, which are of the major relevance to optimize the reaction in the laboratory. In order to demonstrate the robustness of the model we generated a new set of reactions with “perturbations” in the values of T, t, Load, etc. and retrained the models. The values of the values of T, t, Load, where changed randomly but inside the limits of min and max reported for this reactions. This allowed to test the robustness of the model in terms of ability of the model to continue working properly (giving good predictions) despite of changes/errors etc. in the reports of temperature, time, etc.

For this purpose, we generated a new set of reactions with “perturbations” in the values of T (ºC), t(h), Load(%), etc*.* and retrained the models. The values of T (ºC), t(h), Load(%) where changed randomly between the limits set in the minimum V_k_(c_qi_)_min_ and maximum V_k_(c_qi_)_max_ reported for this type of reactions. The synthetic data allow to test the robustness of the PTML model in terms of ability to continue giving good predictions despite of changes/errors, etc*.* In addition, the values of minimum V_k_(c_qi_)_min_, maximum V_k_(c_qi_)_max_, and step V_k_(c_qj_)_step_ for all the operational conditions were calculated (Table 2). Afterwards, we used a MC model based on the following system of equations in order to create the new synthetic data.

**Table 2:** Summary of basic statistics for reactions in the dataset

Stat.^a^	Dataset reaction conditions (c_qi_)^b^		
	T (^o^C)	T (h)	Load (%)
N_reacc_	12	53	7
Avg	11.59	35.87	9.10
S.D	26.80	33.94	5.72
Min	− 78.00	1.00	2.00
Max	66.00	240.00	30.00
Range	144	239	28
Step	10	1	1
N_expr_	14	239	28

^a^Stat. = Statistical parameters for the input parameters (operational conditions) of all the reactions present in our dataset: N_reacc_ = Number of reactions present in our dataset, Avg. = average value, S.D. = Standard deviation, Max. = *maximum* value, Min. = *minimum* value, Range = Max.—Min., Step = minimal change allowed in one experimental condition, N_expr_. = Number of experiments (reactions) changing one condition and keeping the others constant

^b^Operational conditions: T(^o^C) = temperature, t(h) = reaction time, Load(%) = catalyst loading

Firstly, the Eqs. 8 and 9 were applied so as to generate new V_k_(c_qi_)_new_ values starting from the original minimum value V_k_(c_qi_)_min_ (Eq. 8). Later, with the Eq. (9), we obtained the new synthetic data value V_k_(c_qi_)_synth_ after introducing a boundary condition. This boundary condition is set up taking into consideration the conditions of α-amidoalkylation reactions. In other words, the boundary condition keeps the synthetic values V_k_(c_qi_)_synth_ within the range [V_k_(c_qi_)_min_, V_k_(c_qi_)_max_]. The synthetics values were created for the experimental condition variables V_1_(c_qi_) = T(°C), V_2_(c_qi_) = t(h), V_3_(c_qi_) = L(%). It means that the new synthetic data values are equal to V(c_k_)_synth_ = V(c_k_)_min_ + rnd(0, N_max_)·V(c_k_)_step_ iff (if and only if) they are lower than V_k_(c_qi_)_max_; otherwise, they are equal to V_k_(c_qi_)_max_. The function Rnd(0, n_max_) is a generator of pseudo-random natural numbers (n = 0, 1, 2, … N_max_) based on Mersenne-Twister MC algorithm (MT19937). The same system of equations was used to form new synthetic data for the input variables of the reference V_k_(c_rj_) equation.

8 $${{V}_{k}\left({c}_{qi}\right)}_{new}=\left({{V}_{k}\left({c}_{qi}\right)}_{min}+Rnd\left(0,{n}_{max}\right)\cdot {{V}_{k}\left({c}_{qi}\right)}_{step}\right)$$

9 $${{V}_{k}\left({c}_{qi}\right)}_{synth}=if\left[{{V}_{k}\left({c}_{qi}\right)}_{new}>{{V}_{k}\left({c}_{qi}\right)}_{max};{{V}_{k}\left({c}_{qi}\right)}_{max};{{V}_{k}\left({c}_{qi}\right)}_{new}\right]$$

As mentioned above, we have only made small random changes to the values of the input variables t, T, and catalyst loading from the original ones. Consequently, in the new synthetic data cases generated by MC, we assumed that the deviations in the new values of input variables (perturbations) from the original ones are small enough to cause unetectable/non-measurable changes in the output values of *ee*_R_(%). The supposition is based on practical empiric evidence, which seems to confirm that new reactions/repetitions carried out with small changes of a few degrees of Temperature, minutes of reaction time, or catalyst loading will not alter i the value of *ee*R(%) by a measurable amount. In fact, in Eq. (8) the new synthetic value is equal to the minimum value in all the dataset plus the value of the step multiplied by a random value getting values 0, 1, 2, n_max_.

### Experimental methods

We describe here the typical procedure for the enantioselective intermolecular α-amidoalkylation reaction leading to the synthesis of ( +)-**2e** (See Table 8, entry 8). For full experimental details and characterization data for compounds **2a-d**, See Supporting Information file SI00.pdf).

*(* +*)-(R)-2,3-dimethoxy-12b-(1H-pyrrol-2-yl)-5,12b-dihydroisoindolo[1,2-a]isoquinolin-8(6H)-one(****2e****)*. A solution of 12b-hydroxyisoindoloisoquinoline **1** (60 mg, 0.19 mmol), pyrrole **3e** (0.014 mL, 0.19 mmol) and *N*-triflylphosphoramide **4a** (28 mg, 0.038 mmol 20 mol%) in dry THF (5 mL) were stirred during 5 h at room temperature. The solvent was evaporated under reduced pressure, and the crude reaction mixture was purified by flash column chromatography (alumina, Hexane/EtOAc 3:7) to afford isoindolo[1,2-*a*]isoquinoline **2e** (68 mg, quant.); [α]_D_^20^ =  + 40.3 (c = 0.28; CH_2_Cl_2_). The enantiomeric excess was determined by HPLC to be 54% [Chiralcel OD, 15% Hexane/2-propanol, 1 mL/min, t_R_ (*S*) = 23.2 min (22.87%), t_R_ (*R*) = 29.4 min (77.13%)]. m.p. (Hexane/EtOAc): 254–256 °C; IR (Film): 3188 (NH) cm^−1^, 1672 (CO) cm^-1^; ^1^H NMR (300 MHz, CDCl_3_): *δ* 2.70–2.76 (m, 1H), 3.06 (ddd, *J* = 17.3, 11.1, 6.5 Hz, 1H), 3.23 (ddd, *J* = 12.6, 11.1, 4.8 Hz, 1H), 3.85 (s, 3H), 3.87 (s, 3H), 4.26 (ddd, *J* = 12.6, 6.5, 2.2 Hz, 1H), 5.86–5.88 (m, 1H), 6.08 (dd, *J* = 5.8, 2.7 Hz, 1H), 6.62 (s, 1H), 6.74 (td, *J* = 2.7, 1.5 Hz, 1H), 7.23 (s, 1H), 7.44 (t, *J* = 7.5 Hz, 1H),7.58 (t, *J* = 7.5 Hz, 1H), 7.70–7.72 (m, 2H), 8.70 (s, 1H)ppm; ^13^C[^1^H] NMR (75.5 MHz, CDCl_3_):*δ* 28.7, 35.2, 55.9, 56.2, 65.7, 108.1, 110.5, 110.8, 111.7, 119.0, 123.7, 123.9, 127.1, 127.9, 128.8, 131.5, 132.1, 147.1, 148.6, 148.9, 167.2 ppm; MS (CI) m/z (%): 361 (100) [MH]^+^, 360 (50) [M]^+^, 294 (37), 293 (33); HRMS (CI): cacld. for C_22_H_21_N_2_O_3_ [MH]^+^: 361.1552; found: 361.1556.

## Results and discussion

### CPA catalyzed α-amidoalkylation reactions chemical space

As stated above, the chemical space of α-amidoalkylation reactions is very wide. In this work, the dataset is based on 332 reactions which contains 55 different substrates (cyclic and bicyclic hydroxylactams), 53 nucleophiles (enamides, indoles, etc.), 39 chiral catalysts (phosphoric acids, phosphoramides, etc.), and 17 different solvents undertaken by multiple experimental conditions (see Supporting Information, file SI00.pdf for structures and reaction schemes; see Additional file 3 for full details of each reference reaction, including reaction conditions, yield, enantiomeric excess, and SMILE codes for reactants and catalysts in each case). The combination of all possible substrates, catalysts, and reactions conditions to be explored is potentially high to be covered by trial and error experiments. To better understanding the amount of all possible combination, we illustrate an example, if reactions are run independently by changing one reactant at a time, a total of N_comb_ = N(Subs_qi_)·N(Nuc_qi_)·N(Cat_qi_)·N(Solv_qi_) = 55·53·39·17 = 1,932,645 unique combinations of molecule subtypes should be run. This could be a new source of interesting products [changes in N(Subs_qi_) or N(Nuc_qi_)] or a way to improve the reaction efficiency [changes in N(Cat_qi_) or N(Solv_qi_)]. This estimation considers only the combinations of different molecular entities. Unfortunately, the vast majority of these reactions remain unexplored in terms of high cost in time and resources.

On the other hand, there are also important variations in the three main experimental condition variables V_k_(c_qi_) [T(^o^C), t(h), and L(%)]. Table 2 shows different statistics parameters of these variables for the reported reactions. The integer values for maximum (T_max_, t_max_, and L_max_), minimum (T_min_, t_min_, and L_min_), and step (T_step_, t_step_, and L_step_) are included. This is important because the expression Range [V_k_(c_qi_)] = V_k_(c_qi_)_max_ – V_k_(c_qi_)_min_] gives us the range of this variable that can be covered in actual practice in the laboratory. Consequently, when this range is divided by the minimum value, we decided to change in practice Step [V_k_(c_qi_)], the number of experiments N(c_qi_) = Range[V_k_(c_qi_))/Step(V_k_(c_qi_)] that we can run in order to explore this variable can be obtained. When reactions are run independently by changing one experimental condition at a time, a total of N_exp_ experiments must be run. This will be equal to N_exp_ = N(c_1_)·N(c_2_)·N(c_3_) = N(T)·N(t)·N(L) = [Range(T)/Step(T)]·[Range(t)/Step(t)]·[Range(L)/Step(L)] = [144/10]·[(239/1]·[(28/1] = 96,365 optimization experiments for each unique combination of molecule sub-types giving as result an specific Product_qi_ of the reactions R_qi_ (Table 2). The multiplication of both parts of the equation gives an estimate of the very large number of reactions accessible in this chemical space N(R_qi_)_max_ = N_comb_·N_exp_ ≈ 10^11^.The equations used to carry out the calculations of the number of reactions in this chemical space are shown below (Eq. 10) [39]:

10 $${N({R}_{qi})}_{max}=N({Sub}_{qi})\cdot N({Nuc}_{qi}) \cdot N({Cat}_{qi})\cdot N({Solv}_{qi})\cdot \frac{Range\left(T\right)}{Step\left(T\right)}\cdot \frac{Range\left(t\right)}{Step\left(t\right)}\cdot \frac{Range\left(L\right)}{Step\left(L\right)}$$

$${N({R}_{qi})}_{max} =\prod_{s=1}^{s=4}\left[N({m}_{sqi})\right]\cdot \prod_{k=1}^{k=3}\left[\frac{{{V}_{k}({c}_{qi})}_{max}-{{V}_{k}({c}_{qi})}_{min}}{Step({{V}_{k}\left({c}_{qi}\right)}_{max})}\right]$$

$${N({R}_{qi})}_{max}=\prod_{s=1}^{s=4}\left[N\left({m}_{sqi}\right)\right]\cdot \prod_{k=1}^{k=3}\left[N\left({c}_{qi}\right)\right]$$

$${N({R}_{qi})}_{max}={N}_{comb}\cdot {N}_{exp}$$

The previous calculation gives an idea on the dimension of chemical reaction space for enantioselective CPA-catalyzed intermolecular α-amidoalkylation reactions. It is inviable to study all possible combinations in the laboratory due to the time and cost in material and human resources. In the daily practice, chemists can use expert criteria and experimental design techniques to reduce the number of combinations to be tested, to decrease the range of the different experimental conditions variables, etc. This can support researchers to reduce meaningfully the number of reactions to perform in the practice. However, the use of the previous well-known experimental expert criteria, researchers will never test interesting products. Therefore, the main objective of this project was the development of a new user-friendly predictive regression model for these reactions. This predictive model may become a useful tool to reduce the time and cost of experimentation.

### ML linear model for α-amidoalkylation reactions

In the α-amidoalkylation reactions, there is no clear relationship between the chirality of the catalysts and the CIP notation of the product. In fact, in our literature dataset one can note the following ratio of Catalyst/Product chirality relationship, count, and ratio (*R*)*/*(*R*)140 reactions (43.2%), (*S*)*/*(*R*)102 reactions (31.5%), (*R*)*/*(*S*) 72 reactions (22.2%) and (*S*)*/*(*S*) 9 reactions (2.8%) of 324 reactions. There is only one reaction in the entire dataset with an (*S)*configuration catalyst and enantiomeric excess equal to zero. Therefore, it is very important to have a computational model to predict the absolute stereochemistry and the enantiomeric excess of the reaction product. This type of models could be used as a useful tool in order to address the design of new catalysts and/or selecting the optimal reaction conditions a priori. In this work, we decided to tackle this problem using AI/ML techniques. We trained this classic linear ML model using only the Original Data (OD) from reactions. The equation of this model is shown in Eq. 11;

11 $${ee}_{R}{\left(\%\right)}_{qpred} =912.48\cdot Load\left(\%\right)+21.90\cdot T{(}^{o}C) -194.76\cdot t\left(h\right)- 13.21\cdot {\propto \left({Cat}_{qi}\right)}_{Cuns}- 45.02\cdot {\propto \left({Prod}_{q}\right)}_{HetNoX} + 830.06\cdot {\Delta EA\left({Prod}_{q}\right)}_{Csat}-0.34\cdot {EA\left({Cat}_{qi}\right)}_{HetNoX}+ 0.22\cdot {\chi \left({Nuc}_{qi}\right)}_{Het}-2024.05\cdot {\chi \left({Cat}_{q}\right)}_{HetNoX}- 178.69\cdot {V\left({Sub}_{qi}\right)}_{Tot} - 1678.05\cdot {Zv\left({Cat}_{qi}\right)}_{Cuns}- 34.41\cdot {Zv\left({Solv}_{q}\right)}_{Cuns} -0.70$$

$$n=332(reactions) {R}^{2}=0.74 F= 59.2 p<0.5$$

This ML model does not use reference reactions for comparison. The statistic parameters of the model are n = 332, Regression coefficient R^2^ = 0.74, Fisher ratio F = 59.2, Standard Error of Estimates SEE = 37.1, p-level p < 0.05. More detailed information about coefficients and variables of the model as well as symbols and names of variables, Standard Error (SE), Students’ t values, and p-level are given in Table 3. The model obtains 74.0% of variance (coefficient R^2^ = 0.74), which is an acceptable prediction percentage for organic synthesis reactions (although extremely improbable). By the way, the SEE = 37.1 could be considered relatively high[39]. On the other hand, an essential short-coming of this classic ML linear model is that it does not provide us any evidence about the most similar reactions conveyed in the scientific literature. Consequently, this may limit our ability to deduce possible mechanisms and/or compare our results with others already known. Therefore, this ML model needs to be used along with another search strategy for similar molecules to obtain clues of similar reactions for a specific reaction under study. One option is to couple this model with similarity search strategies based on Tanimoto’s similarity indices [43]. In fact, there are interesting works that report the coupling of Cheminformatics models with search strategies based on similarity [44–46]. A well-known example of online search tools is the Scifinder platform [47, 48].

**Table 3:** Results of the PTML regression model

Model	Input Vars^a^	Symbol	Coeff	*ee*_R_(%)_qr_^b^	S.E.^c^	t^d^	p-level^e^
ML	Load(%)_qr_	V_3_(c_qi_)	a_1_	912.48	258.3259	3.53227	< 0.05
	T(^o^C)_qr_	V_1_(c_qi_)	a_2_	21.90	18.7647	1.16732	0.24
	t(h)_qr_	V_2_(c_qi_)	a_3_	− 194.76	55.2274	− 3.52654	< 0.05
	α(Cat_qi_)_Cuns_	D_4_(m_3qi_)_2_	b_1_	− 13.21	2.7499	− 4.80465	< 0.05
	α(Prod_qi_)_HetNoX_	D_4_(m_5qi_)_4_	b_2_	− 45.02	21.8624	− 2.05905	< 0.05
	EA(Prod_qi_)_Csat_	D_5_(m_5qi_)_1_	b_3_	830.06	163.3526	5.08139	< 0.05
	EA(Cat_qi_)_HetNoX_	D_5_(m_3qi_)_4_	b_4_	− 0.34	0.0949	− 3.62574	< 0.05
	χ(Nuc_qi_)_Het_	D_3_(m_2qi_)_3_	b_5_	0.22	0.0742	3.01949	< 0.05
	χ(Cat_qi_)_HetNoX_	D_3_(m_3qi_)_4_	b_6_	− 2024.05	484.4448	− 4.17809	< 0.05
	V(Sub_qi_)_Tot_	D_2_(m_1qi_)_5_	b_7_	− 178.69	43.4355	− 4.11390	< 0.05
	Zv(Cat_qi_)_Cuns_	D_1_(m_3qi_)_2_	b_8_	− 1678.05	468.7747	− 3.57965	< 0.05
	Zv(Solv_qi_)_Cuns_	D_1_(m_4qi_)_2_	b_9_	− 34.41	11.6896	− 2.94399	< 0.05
	Intercept	–	e_0_	− 0.70	0.5150	− 1.35948	0.18

^a^Input variables with coefficient b_k_ are the values of shift (Δ) in q-reac *vs*. r-reac for different properties: α = average atomic polarizability, EA = average atomic Electro Affinity, χ = average atomic Sanderson Electronegativity, Zv = average atomic number

^b^Coefficients of the variables in the model, the output variable is the Δ in enantiomeric excess *ee*(%)^*^ of the q-reac with respect to the r-reac when both reactions have been carried out with(*R*)-catalyst

^c^Standard error of the coefficients

^d^Student t-value

^e^p-level of error

### PTML model for α-amidoalkylation reactions

**Fig. 1:**
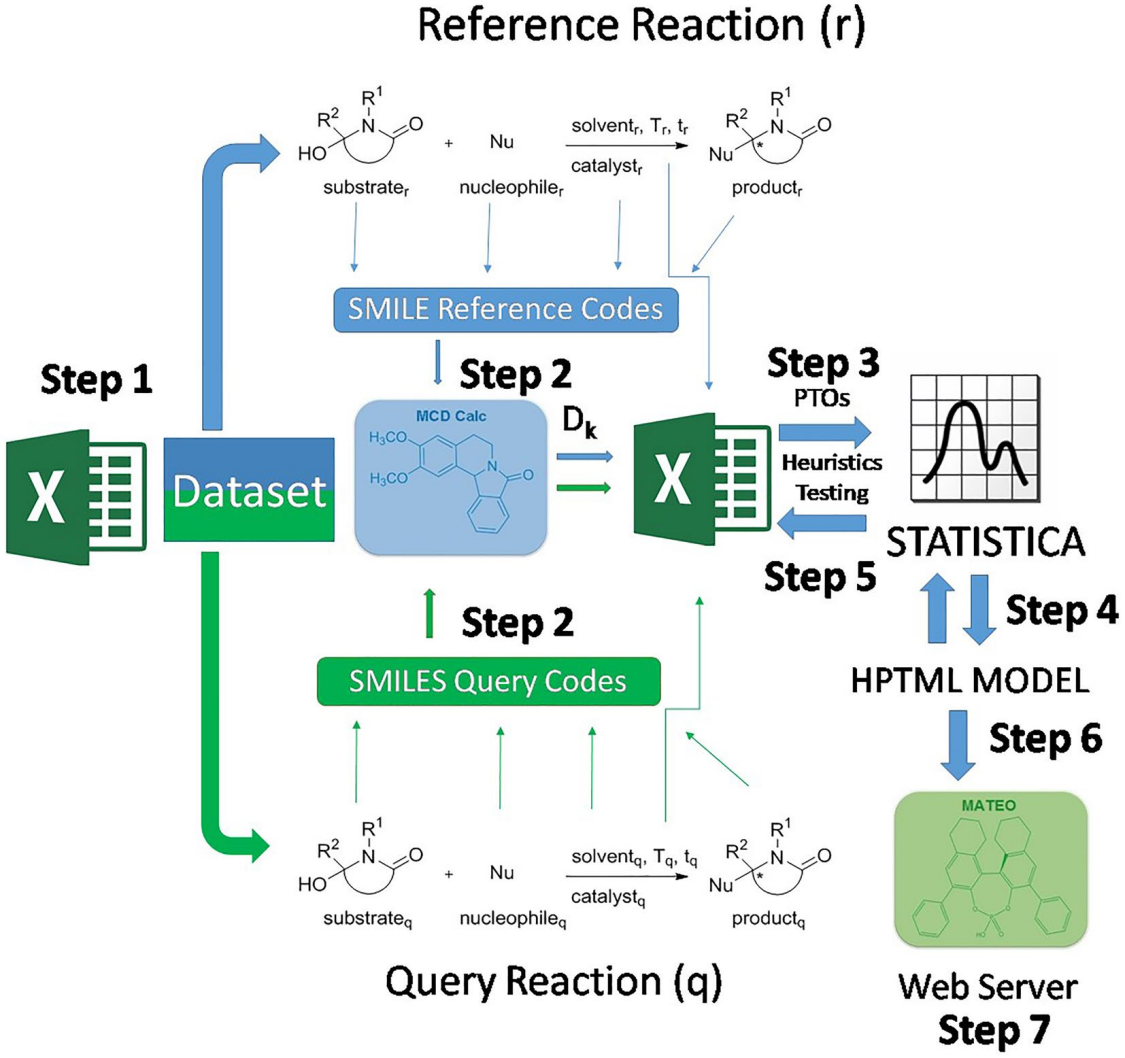
HPTML models general workflow used in this work

As mentioned in the previous section, we have reported a PTML model for α-amidoalkylation reactions, although it is difficult to use in practice and not implemented on a publicly available online web server. Unfortunately, the input variables used in that model are not available as an open source code. For this reason, it could be advantageous to implement the model on a public online server. Consequently, we decided to develop a new linear PTML model using our own library to calculate the molecular descriptors. PTML reactivity models can study pair-wise reactions [39]. The model infers the reactivity of a query reaction (q) by comparing it to a previously known reference reaction (r). Some PTML models use different Heuristics (H) to match q and r reactions. These models can be called HPTML models. The Fig. 1 illustrates the general workflow that has been followed during this word to look for the new HPTML models. In step 1, the reference dataset and reaction pairs q *vs*. r were created. In step 2, the SMILE codes of the molecules (m_qsi_, m_rsj_) involved in both q and r reactions (substrates, nucleophiles, catalysts, solvents, products) were entered in the MCDCalc server [49] to calculate their molecular descriptors D_k_(m_qsi_)_g_ and D_k_(m_rsj_)_g_. In step 3, the PTOs for pairs of reactions were calculated. In step 4, the Multivariate Linear Regression (MLR) algorithm implemented in the STATISTICA [38] software was used to seek the PTML model. In step 5, heuristics H_1_ and H_2_ were tested interactively. In step 6, the best HPTML model was selected. Finally, in step 7, this model was implemented on a public web server (see the following sections). The best linear HPTML model found is shown in Eq. 12;

12 $${\Delta ee}_{R}{\left(\%\right)}_{qr}=-0.82\cdot \Delta Load\left(\%\right)-0.34\cdot \Delta T{(}^{o}C)+0.21\cdot \Delta t(h)-174.37\cdot {\Delta \propto \left({Cat}_{q},{ Cat}_{r}\right)}_{Cuns} -1534.17\cdot {\Delta \propto \left({Prod}_{q},{ Prod}_{r}\right)}_{HetNoX} -215.98\cdot {\Delta EA\left({Prod}_{q},{ Prod}_{r}\right)}_{Csat} -1747.12\cdot {\Delta EA\left({Cat}_{q},{ Cat}_{r}\right)}_{HetNoX} -42.49\cdot {\Delta \chi \left({Nuc}_{q},{ Nuc}_{r}\right)}_{Het}+750.76\cdot {\Delta \chi \left({Cat}_{q},{ Cat}_{r}\right)}_{HetNoX} -34.19\cdot {\Delta V\left({Sub}_{q},{ Sub}_{r}\right)}_{Tot}+22.04\cdot {\Delta Zv\left({Cat}_{q},{ Cat}_{r}\right)}_{Cuns} -12.46\cdot {\Delta Zv\left({Solv}_{q},{ Solv}_{r}\right)}_{Cuns} -0.91$$

$$n=78732 (react. pairs) {R}_{train}=0.84 F= 15238.7 p<0.5$$

The HPTML model was trained with a total of n_train_ = 78,732 arbitrarily selected reaction pairs. The statistical parameters obtained for this model are the regression coefficient value of R_train_ = 0.84 and Standard Error of Estimates SEE = 51.67 and a Fisher’s ratio of F = 15,238.7 with a p-level < 0.05 in training series. This points out a important relationship between the observed relative values of ∆*ee*_R_(%)_qrobs_ and the predicted values ∆*ee*_R_(%)_qrobs_.

In addition, another subset of n_val_ = 28,836 reaction pairs was used to validate the model. A regression coefficient R_val_ = 0.77 and SEE = 60.225 were found for this validation series. The output of the model is *ee*_*R*_(%)_qrcalc_. This variable represents the enantiomeric excess value calculated using a single reference reaction. The *ee*_*R*_(%)_calc_ value quantifies the enantiomeric excess obtained using an (*R*)-catalyst. If *ee*_R_(%)_calc_ > 0, the product is predicted to have (*R*) notation; if *ee*_R_(%)_calc_ < 0, the product is predicted to have (*S*) notation; if *ee*_R_(%)_calc_ = 0 racemic mixture. The overall p-level of the model is p < 0.05. All the variables introduced in the model are statistically significant (Table 4**)**. The three first input variables quantify the effect of non-structural factors on the enantioselectivity parameter, *ee*_R_(%)_calc_. The remaining input variables quantify the contribution of structural variations in the Substrate (Sub), Catalyst (Cat), Product (Prod), Nucleophile (Nuc), and Solvent (Solv).

**Table 4:** Results of the PTML regression model

Model	Input Vars^a^	Symbol	Coeff.^b^	Δ*ee*_R_(%)_qr_^b^	S.E.^c^	t^d^	p-level^e^
PMTL	ΔLoad(%)_qr_	ΔV_3_(c_qi_, c_rj_)	a_1_	− 0.82	0.03243	− 25.3154	< 0.005
	ΔT(oC)_qr_	ΔV_1_(c_qi_, c_rj_)	a_2_	− 0.34	0.00594	− 57.8960	< 0.005
	Δt(h)_qr_	ΔV_2_(c_qi_, c_rj_)	a_3_	0.21	0.00476	44.4627	< 0.005
	α(Cat_q_, Cat_r_)_Cuns_	ΔD_4_(m_3qi_, m_3rj_)_2_	b_1_	− 174.37	2.67954	− 65.0741	< 0.005
	α(Prod_q_, Prod_r_)_HetNoX_	ΔD_4_(m_5qi_, m_5rj_)_4_	b_2_	− 1534.17	26.38185	− 58.1525	< 0.005
	EA(Prod_q_, Prod_r_)_Csat_	ΔD_5_(m_5qi_, m_5rj_)_1_	b_3_	− 215.98	3.38484	− 63.8086	< 0.005
	EA(Cat_q_, Cat_r_)_HetNoX_	ΔD_5_(m_3qi_, m_3rj_)_4_	b_4_	− 1747.12	26.48292	− 65.9715	< 0.005
	χ(Nuc_q_, Nuc_r_)_Het_	ΔD_3_(m_2qi_, m_2rj_)_3_	b_5_	− 42.49	1.33694	− 31.7788	< 0.005
	χ(Cat_q_, Cat_r_))_HetNoX_	ΔD_3_(m_3qi_, m_3rj_)_4_	b_6_	750.76	8.98832	83.5259	< 0.005
	V(Sub_q_, Sub_r_)_Tot_	ΔD_2_(m_1qi_, m_1rj_)_5_	b_7_	− 34.19	0.70023	− 48.8225	< 0.005
	Zv(Cat_q_, Cat_r_)_Cuns_	ΔD_1_(m_3qi_, m_3rj_)_2_	b_8_	22.04	1.16398	18.9356	< 0.005
	Zv(Solv_q_, Solv_r_)_Cuns_	ΔD_1_(m_4qi_, m_4rj_)_2_	b_9_	− 12.46	0.16653	− 74.8101	< 0.005
	Intercept	–	e_0_	− 0.91	0.18257	− 5.0065	< 0.005

^a^Input variables with coefficient b_k_ are the values of shift (Δ) in q-reac*vs*. r-reac for different properties: *α* average atomic polarizability, *EA* average atomic Electro Affinity, *χ* average atomic Sanderson Electronegativity, *Zv* average atomic number

^b^Coefficients of the variables in the model, the output variable is the Δ in enantiomeric excess *ee*(%)^*^ of the q-reac with respect to the r-reac when both reactions have been carried out with(*R*)-catalyst

^c^Standard error of the coefficients

^d^Student t-value

^e^p-level of error

### PTML calculations with a single reference reaction

As we explained above, this PTML reactivity model studies pair-wise reactions. To avoid distortions in the distribution of the variables, PTML model uses the variable ∆*ee*_R_(%)_qrobs_ as objective function (see Eq. 5) [39]. This objective function is the function to fit and is equal to ∆*ee*_R_(%)_qrobs_ = *ee*_R_(%)_qobs_—*ee*_R_(%)_robs_. As a result, the output of the new model is ∆*ee*_R_(%)_qrcalc_ = *ee*_R_(%)_qcalc_- *ee*_R_(%)_rcalc_. For non-accurate models ∆*ee*_R_(%)_qrcalc_ ≠ ∆*ee*_R_(%)_qrobs_ (where ≠ indicates not ≈). Conversely, for a not-random accurate predictor, like this one, one can approximate ∆*ee*_R_(%)_qrcalc_ ≈ ∆*ee*_R_(%)_qrobs_. This presupposes that *ee*_*R*_(%)_qcalc_ ≈ *ee*_R_(%)_qobs_ and *ee*_*R*_(%)_rcalc_ ≈ *ee*_R_(%)_robs_. Therefore, for practical purposes, we use the model to predict the enantiomeric excess of new query reactions *ee*_*R*_(%)_qcalc_, based on the observed enantiomeric excess of a reference reaction *ee*_*R*_(%)_qrobs_. The approximation is only valid for not-random accurate predictors and takes into account that *ee*_*R*_(%)_rcalc_ ≈ *ee*_*R*_(%)_robs_ is always a known reference reaction, so it is necessary to rearrange the variables in Eq. 5 as shown in Eq. 13;

13 $${ee}_{R}{\left(\%\right)}_{calcqi}={ee}_{R}{\left(\%\right)}_{refj}-0.82\cdot \Delta Load\left(\%\right)-0.34\cdot \Delta T{(}^{o}C)+0.21\cdot \Delta t(h) -174.37\cdot {\Delta \propto \left({Cat}_{q},{ Cat}_{r}\right)}_{Cuns} -1534.17\cdot {\Delta \propto \left({Prod}_{q},{ Prod}_{r}\right)}_{HetNoX} -215.98\cdot {\Delta EA\left({Prod}_{q},{ Prod}_{r}\right)}_{Csat} -1747.12\cdot {\Delta EA\left({Cat}_{q},{ Cat}_{r}\right)}_{HetNoX} -42.49\cdot {\Delta \chi \left({Nuc}_{q},{ Nuc}_{r}\right)}_{Het}+750.76\cdot {\Delta \chi \left({Cat}_{q},{ Cat}_{r}\right)}_{HetNoX} -34.19\cdot {\Delta V\left({Sub}_{q},{ Sub}_{r}\right)}_{Tot}+22.04\cdot {\Delta Zv\left({Cat}_{q},{ Cat}_{r}\right)}_{Cuns} -12.46\cdot {\Delta Zv\left({Solv}_{q},{ Solv}_{r}\right)}_{Cuns} -0.91$$

**Fig. 2:**
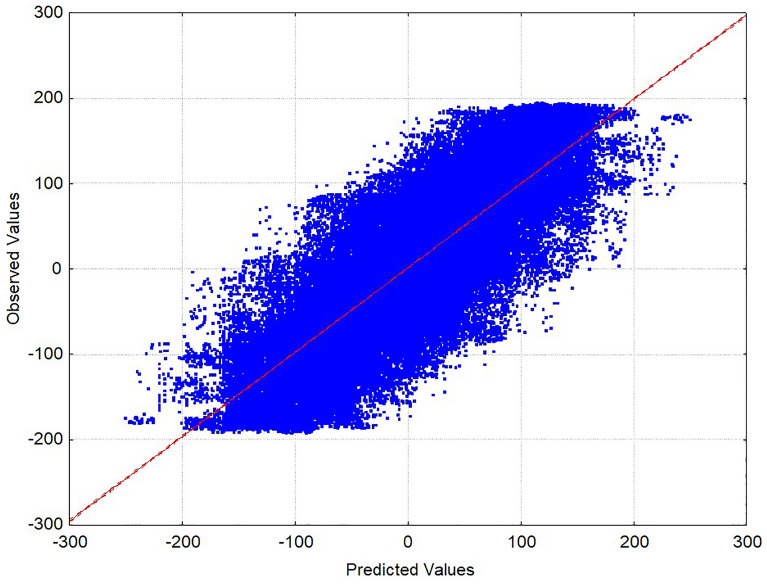
Observed *vs*. Predicted (Δ*ee*_*R*_(%)_qrobs_ *vs*. Δ*ee*_*R*_(%)_qrcalc_) for equation Eq. 12 (R = 0.84 in training series). Only 10,000 reaction pairs of reactions (cases) are depicted due to software limitations

As a result of this approach, the model calculates different values of *ee*_R_(%)_calcqi_ for the same reaction depending on the experimental value *ee*_*R*_(%)_refj_ of the reaction used as reference in the pair [39]. Figure 2 illustrates the observed values of Δ*ee*_*R*_(%)_qrobs_ *vs*. the predicted (calculated) values of Δ*ee*_*R*_(%)_calcqi_ for 10,000 selected reaction pairs. We depict only 10000 pairs due to software plotting limitations (this the top number of points allowed by the software). A certain linear trend is observed (points with ∆*ee*_*R*_(%)_qrcalc_ ≈ ∆*ee*_*R*_(%)_qrobs_), however, despite being a predictor with adequate goodness of fit, there are many points with higher dispersion (points with ∆*ee*_*R*_(%)_qrcalc_ ≠ ∆*ee*_*R*_(%)_qrobs_).

In fact, PTML models may be included on a broader class of learning problems, such as delta ML, transfer ML, template selection ML, etc*.* [50–53]. In general, these models involve the use of a query item (item to be predicted) compared to a reference item (template, pair, known case, item from related domain, etc.). To calculate the output of a query item (quantum field, drug, protein, or reaction in this case), it is necessary to use an already known item or population of reference items as input. Query items can be in the same or a different data domain from the reference item. In this context, the low population (low number of available cases) of some of the studied data subset (data domains) is also a common problem. In our case, to calculate the value of *ee*_R_(%)_calcqi_ for a query reaction (q), the observed *ee*_R_(%)_refj_ values of an already known reference reaction (r) must be used as input. Here both the query and reference items come from the same data domain (both are the same type of reactions). The reaction of reference can be selected from our reaction dataset (same data domain) [54]. Consequently, for a new query reaction, there are n = 332 reactions in the dataset that can be used as the reference reaction, which pave the way for the question of which is/are the best candidate/candidates to be used as reference reaction in each case (see next section). Thus, 332 different values of *ee*_R_(%)_calcqi_ can be calculated for the same query reaction based on the selected pairing reaction of reference. In this step, heuristic rules can be used to approximate the final predicted value *ee*_R_(%)_qpred_ depending on the *ee*_R_(%)_calc_ values of the model, as we have demonstrated previously to solve a similar problem [39].

### HPTML model for prediction with multiple reactions of reference

**Fig. 3:**
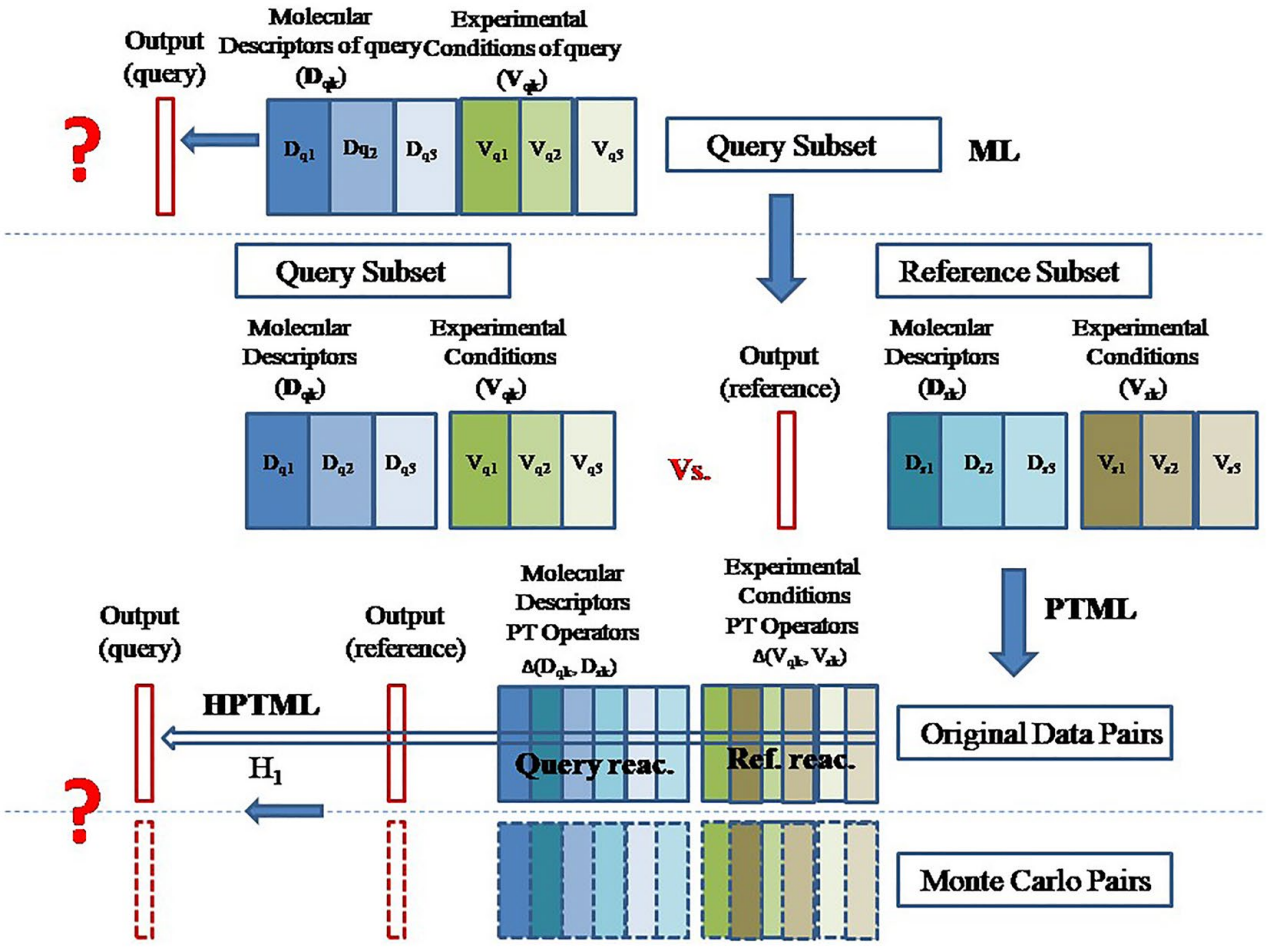
HPTML data re-arrangement and MC data enrichment schematic illustration

As mentioned above, it is necessary to define the best reaction or set of reactions to use. Defining an appropriate reference reaction can also help reduce the dispersion and increase the value of the regression coefficient, because each query reaction will have a single predicted value. With this purpose, a Heuristic rule coupled to the PTML model can be used to select the best reference. Heuristic-based methods have been widely used in Cheminformatics to solve practical problems [55–57]. In our case, the combination of the PTML model with a Heuristic (H) rule defines the term HPTML = H + PTML algorithm. Two Heuristics (H_1_ and H_2_) were tested by calculating the *ee*_R_(%)_qrpred_ values for the 332 reactions in our dataset, using the PTML trained with the OD set. These HPTML models based on Heuristics H_1_ and H_2_ were compared with a classic ML model. This classic ML model includes no PT terms and was built without using Heuristics (H_0_). Figure 3 shows a schematic illustration of the ML, PTML, and HPTML data re-arrangement, as well as the MC data enrichment procedures used here.

Table 5 shows the statistical parameters for these studies (see only entries with Data = OD). Detailed information can be found in Additional file 2: Table S1of the Supporting Information file (Additional file 2). It should be noted that both HPTML models using Heuristics give good results with an OD regression coefficient in the range R^2^ = 0.64–0.81 and p < 0.05. Specifically, the HPTML OD H_1_ model has a higher regression coefficient (R^2^ = 0.81 *vs*. 0.55) and a lower SEE (R^2^ = 29.5 *vs*. 37.1) than the classic ML model. However, this SEE value is still relatively high. Interestingly, MC data enrichment improved both R^2^ = 0.96 and SEE = 13.5 values of the HPTML OD H_1_ model. In addition, the HPTML model automatically provides the most similar reference reaction from the reference dataset, including the reference of the article, which might give some clues about the possible reaction mechanism, etc. of the query reaction. In contrast, the classic ML model does not give information about the plausible reaction mechanism or similar reactions in the literature. Overall, these results justify the use of the HPTML algorithm instead of the classic ML algorithm.

**Table 5:** HPTML models obtained with different datasets *vs*. alternative heuristics

Algorithm^a^	Data	Heuristic	n_reacc_^b^	n_pairs_^c^	R^2^	SEE	F	p-level
ML	OD	H_0_	332	0	0.55	37.1	59.2	< 0.05
HPTML	OD	H_1_	332	107626	0.81	29.5	1332.2	< 0.05
	OD	H_2_	332	107626	0.64	39.3	603.0	< 0.05
HPTML	ODMC	H_1_	332	109298	**0.96**	13.5	7560.6	< 0.05
+ MC	ODMC	H_2_	332	109298	0.66	38.7	631.4	< 0.05

^*a*^*OD* Original Data, *MC* Monte Carlo, *ODMC* OD + MC enriched dataset

^b^*n*_*reacc*_ Number of reactions present in our dataset

^c^*n*_*pairs*_ Number of pairs of reactions present in our dataset

Interestingly, the pair-wise strategy can rapidly increase the number of cases, as you go from datasets with n items (reactions) to n x n items (pairs of reactions). In this case, we go from n_reacc_ = 332 reactions to n_pairs_ = 107,626 pairs of reactions, which could be an advantage of PTML model, since increasing the number of items to train the ML model can improve learning. However, those items that are underrepresented in the original data are still underrepresented in the new data in relative terms. In addition, you take the risk of including mismatched pair, that is, you take the risk of trying to predict an underrepresented query item (reaction) using as reference an overrepresented item (reaction family) that is not similar to the reference. For example, reactions from the ***aaa*** family are generally the most represented with n_reacc_ = 120 cases (36.14% of cases) and n_pairs_ = 37,570 (34.91%) including many pairs with reactions from the same family. In contrast, reactions from the ***dab*** family are very poorly represented (low abundance) with only n_reacc_ = 3 cases (0.9% of cases) appearing in n_pairs_ = 995 pairs of reactions. Almost all of these pairs are formed with reactions from other families and the relative abundance remains low (0.9%).

Table 6 shows the absolute and relative abundance of different reaction families (subsets) in the original dataset and the number of pairs formed with them. It should be noted that the formation of pairs of mismatched reactions can lead to inaccurate predictions. For example, predicting a query reaction from the ***aab*** family may give an inaccurate result if we use a reaction from the ***haa*** family as reference, because ***aab*** reactions have an average enantiomeric excess < *ee*_R_(%) > _qobs_ = 21.0 while ***haa*** reactions have < *ee*_R_(%) > _qobs_ = -78.1. Both reaction families not only have a markedly different average enantiomeric excess, but also give products with reverse (*R*) or (*S*) CIP notation of absolute configuration [31]. The compound codes, SMILE codes, and chemical structures of the different families of substrates, nucleophiles, and catalysts are shown on the Supporting Information file SI00.pdf.

**Table 6:** Selected subsets of reactions

Structural			React	Absolute abundance ^b^			*S*-catalyst products		*R*-catalyst products	
Patterns^a^			Family	OD	OD	MC	< *ee*_S_(%) > _qobs_		< *ee*_R_(%) > _qobs_	
Sub	Nuc	Cat	(Subset)	n_reacc_	n_pairs_	n_mcpairs_	*S*	*R*	*R*	*S*
*a*	*a*	*a*	***aaa***	120	37570	605	− 43.2	76.1	43.2	− 76.1
*a*	*f*	*a*	***afa***	42	13943	211	− 71.0	76.0	71.0	− 76.0
*h*	*a*	*a*	***haa***	38	12616	190	ND	78.1	ND	− 78.1
*c*	*a*	*b*	***cab***	29	9628	145	− 17.0	69.8	17.0	− 69.8
*a*	*f*	*b*	***afb***	19	6307	95	− 53.0	87.4	53.0	− 87.4
*a*	*c*	*b*	***acb***	17	5644	85	ND	50.4	ND	− 50.4
*c*	*a*	*a*	***caa***	14	4648	70	− 15.5	27.7	15.5	− 27.7
*e*	*a*	*b*	***eab***	8	2656	40	ND	79.9	ND	− 79.9
*a*	*a*	*b*	***aab***	4	1328	20	− 21.0	ND	21.0	ND
*d*	*a*	*b*	***dab***	3	995	15	ND	85.7	ND	− 85.7

^*a*^*Sub* Substrate, *Nuc* Nucleophile, *Cat* Catalyst, patterns *a*, *b*, *c*, *aaa*, etc. are the different families of reactants/reactions, see the text, ND no data

^*b*^*OD* Original Data, *MC* Monte Carlo, *ODMC* OD + MC enriched dataset. *n*_*reacc*_ Number of reactions present in our dataset. *n*_*pairs*_ Number of pairs of reactions present in our dataset, *n*_*mcpairs*_ Number of pairs of reactions present in our dataset in MC experiments

**Fig. 4:**
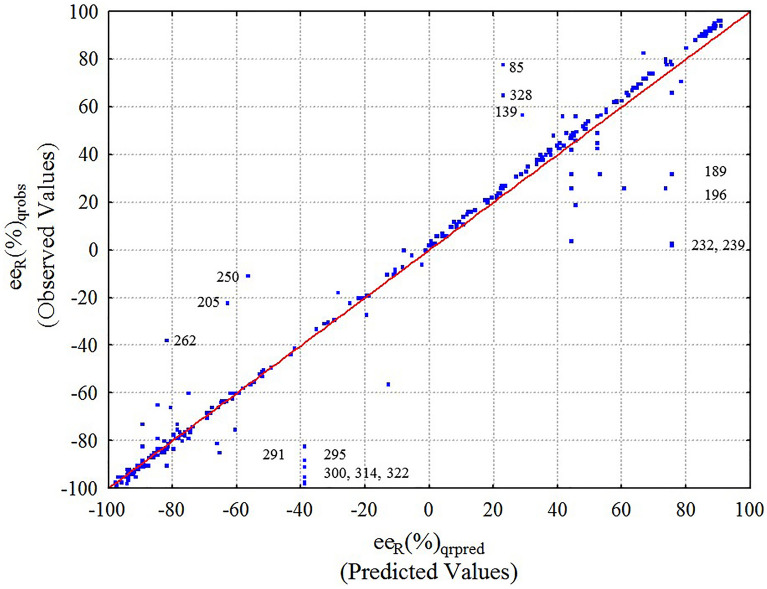
HPTML *ee*_R_(%) observed *vs*. predicted values with Eq. 12 (R^2^ = 0.98) after applying both MC synthetic data and best Heuristic rule (ODMC + H_1_). Overall data for training and validation series. The reaction number from the database (See Additional file 2) has been included for selected examples

In this regard, synthetic data generation techniques can be used to palliate the presence of low populated data subsets. In any case, the total abundance of each enriched data subset should remain essentially constant to avoid creating data artifacts. MC sampling methods have widely used in chemistry for similar purposes [58]. To palliate this situation, we have used a Mersenne-Twister MC algorithm (MT19937) [59] for data enrichment by creating new synthetic data. Therefore, synthetic data cases of the input variables V_k_(c_qi_) = T(°C)_qi_, t(h)_qi_, or L(%)_qi_ of query reactions were generated using a MC algorithm (see system of equations in Materials and Methods section). The same MC algorithm (system of equations) was used to generate new synthetic data for the input variables of the equation of reference V_k_(c_rj_). Nevertheless, the molecular descriptors D_k_(m_sqi_) and D_k_(m_srj_) were never modified in the MC data enrichment simulation, because one can reasonably expect that small changes in the input reaction condition variables [V_k(cqi)_ = T(°C), t(h), or L(%)] do not to significantly change the output *ee*_R_(%). However, the same cannot be guaranteed for changes in chemical structure. Thus, we obtained a slightly higher number of cases for very low abundant reactions. For example, we were able to add n_mcpairs_ = 15, 20, or 40 new cases for the ***dab****, ****aab***, and ***eab*** families of reactions; but we kept their relative abundance essentially low in the range, 0.9–2.47%. Table 6 shows that both models trained with the ODMC dataset (OD enriched by MC) give essentially the same value of R = 0.8–0.9 and p < 0.05 obtained with OD alone. However, the error decreased from SEE = 29.5% to SEE = 13.5% using Heuristic H_1_. Table 7 shows the correlation matrix for the outputs of all models that illustrates the high correlation obtained among them, R = 0.80–0.99. The results of *ee*_R_(%)_qrobs_ observed *vs*. *ee*_R_(%)_qrpred_ predicted with this HTPML model using ODMC dataset and H_1_ heuristic are graphically depicted in Fig. 4, where each point corresponds to a reaction included in the dataset. It can be graphically observed that although an excellent correlation of the predicted and obtained *ee*(%) value is generally obtained, some values are far from the line of correlation. In selected cases, the corresponding reaction number from the database (See SI001.xls file) has been included. It is difficult to draw any conclusions from these cases, as the reactants used are structurally heterogeneous and the experimental conditions diverse as well. In any case, the model has already a very high R^2^ = 0.98 value. We can conclude that using ODMC enriched data decreased the error of the model without decreasing the regression quality.

**Table 7:** HPTML Data set *vs.* heuristics correlation matrix

	HPTML		*ee*_R_(%)_qcalc_				
	Models^a^		OD	OMCD	MCD	OD	MCD
Heuristic	Data	*ee*_R_(%)_qobs_		H_1_		H_2_	
H_1_	OD	0.90	1.00				
	ODMC	0.98	0.92	1.00			
	MC	0.99	0.90	0.98	1.00		
H_2_	ODMC	0.80	0.84	0.81	0.80	1.00	
	MC	0.81	0.84	0.81	0.81	1.00	1.00

^a^*OD* Original Data, *MC* Monte Carlo, *ODMC* OD + MC enriched dataset

### HPTML *vs.* Experimental study of new reactions

In this section, we report an additional test of the HPTML model comparing the computational predictions with the experimental study of new reactions. Thus, we performed both an experimental and a theoretical study of new intermolecular α-amidoalkylation reactions not previously reported in the literature. First, the α-amidoalkylation reactions carried out experimentally are described. Next, we report the use of the HPTML model to predict these reactions and compare the results with the experimental values.

### Experimental study of α-amidoalkylation reactions.

**Scheme 4.**
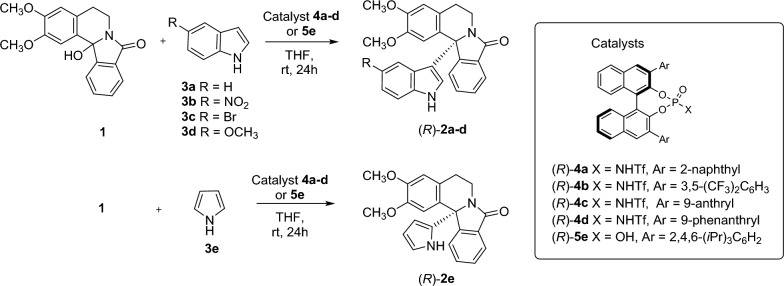
New enantioselective α-amidoalkylation reactions of indoles **3a-d** and pyrrole **3e** with Triflamide catalysts **4a-d** and their comparison with CPA catalyst **5e.** Synthesis of enantioenriched isoindoloisoquinolines **2a-e** (Table 8)

As stated above, the α-amidoalkylation reaction is a very attractive method for C–C bond formation in organic synthesis. In this context, we have previously reported [27] that the α-amidoalkylation reaction is an efficient procedure for the enantioselective synthesis of 12b-substituted isoindoloisoquinolines (Nuevamine-type alkaloids [60]) using BINOL-derived Brønsted acids as catalysts. It should be pointed out that these catalysts have been used in intermolecular α-amidoalkylation of indoles with cyclic *N-*acyliminium ions formed in situ from cyclic hydroxylactams to form tertiary or quaternary stereogenic centers, but this was the first example of bicyclic *N*-acyliminium intermediates in intermolecular α-amidoalkylation reactions of indoles [30].The best results were obtained using a sterically demanding CPA (20 mol% catalyst loading) under the following conditions: THF as solvent at room temperature for 24 h. However, in some cases, moderate enantioselectivity (enantiomeric excess) and/or yields were obtained. Therefore, we decided to test BINOL-derived *N*-triflylphosphoramides as catalysts to enhance the enantioselectivity of these reactions, because they are known to have an increased acidity when compared to the corresponding CPAs, so they can form tighter ion pairs leading to an improved reactivity [61, 62]. Thus, the *N*-triflylphosphoramides **4a**-**d** were synthesized [63, 64] and tested as catalysts in the reaction of 12b-hydroxyisoindoloisoquinoline **1** with the indoles **3a**-**d** (Scheme 4). Table 8 summarizes these new results compared with those previously obtained with phosphoric acid **5e**, which has demonstrated to be the most efficient catalyst for indole [30].The best results were obtained with the catalyst **4a**, although good to excellent yields were achieved with all the phosphoramides. Successfully, we were able to improve our previous result obtaining with the corresponding phosphoric acids, obtaining **2a** with excellent yield and enantioselectivity (90, 93% *ee*). In addition, the intermolecular α-amidoalkylation reaction was extended to 5-substituted indoles **3b**-**d**, obtaining excellent yields, even when a strong acceptor group (NO_2_) was introduced (Table 8, entry 5). However, the use of the substituted indoles led to lower enantiomeric excesses (Table 8, entries 5–7). The reaction could also be applied to other electron-rich heteroaromatics as pyrrole **3e**, obtaining **2e** quantitatively, although with moderate *ee* (Table 8, entry 8). In this case, the reaction was cleaner and faster (reaction completed in 5 h) than when using phosphoric acid **5e** as catalyst (Table 8, entries 13–15).

**Table 8:** Enantioselective intermolecular α-amidoalkylation reactions of *N*-triflamides *vs*. phosphoric acids as catalysts

Reactions	entry	Nuc_qi_	Cat_qi_	Prod	Yield (%)^a^	*ee*_*R*_(%)_obs_^b^
New reactions (Catalysts **4a**-**4d**)	1	**3a**	**4a**	**2a**	90	93
	2	**3a**	**4b**	**2a**	70	0
	3	**3a**	**4c**	**2a**	70	26
	4	**3a**	**4d**	**2a**	70	65
	5	**3b**	**4a**	**2b**	94	11
	6	**3c**	**4a**	**2c**	quant	67
	7	**3d**	**4a**	**2d**	quant	52
	8	**3e**	**4a**^c^	**2e**	quant	54
Reported reactions (Catalyst **5e**)	9	**3a**	**5e**^d^	**2a**	70	74
	10	**3b**	**5e**^d^	**2b**	–	–
	11	**3c**	**5e**^d^	**2c**	74	58
	12	**3d**	**5e**^d^	**2d**	79	74
	13	**3e**	**5e**	**2e**	77	21
	14	**3e**	**5e**^e^	**2e**	18	12
	15	**3e**	**5e**^f^	**2e**	27	24

^a^Yield (%) of isolated pure compound, the symbols **2a** - **5e** are the reactants and products, see scheme 4

^b^Determined by chiral stationary-phase HPLC

^c^Reaction time: 5 h

^d^Reactions previously reported by our group using the (*R*)-phosphoric acid **5e** as catalyst

^e^Temperature: − 30 ºC

^f^ Catalyst loading 2.5 mol%, reaction time 48 h

### HPTML prediction of new α-amidoalkylation reactions

Next, using the developed HPTML ODMC H_1_ model, we predicted the values of *ee*_R_(%) for the new enantioselective intermolecular α-amidoalkylation reactions. We first calculated the molecular D_k_(m_qsi_)_g_ descriptors of all the molecules (Substrate_qi_, Nucleophile_qi_, Catalyst_qi_, Solvent_qi_, and Product_qi_) involved in the new query reactions (R_q_) using the web server MCDCalc [38]. Then, the Heuristic H_1_was used to find the best reference reaction for each new query reaction. Next, we substituted in the model equation the values of the molecular descriptors D_k_(m_qsi_)_g_ and D_r_(m_rsj_)_g_ of the molecules, as well as the values of the input experimental conditions variables V_k_(c_qi_) and V_k_(c_rj_), from both the query (R_q_) and reference reaction (R_r_), respectively. Table 9 shows the predicted *ee*_R_(%) values for each reaction compared to the values predicted with the other Datasets (OD *vs*. ODMC) and Heuristics (H_1_ and H_2_).

**Table 9:** HPTML study of new enantioselective intermolecular α-amidoalkylation reactions

Reaction inputs^a^		Reaction features^b^	New reactions (Table 8)							
			1	2	3	4	5	6	7	8
Reactants		Nuc	**3a**	**3a**	**3a**	**3a**	**3b**	**3c**	**3d**	**3e**
		Cat	**4a**	**4b**	**4c**	**4d**	**4a**	**4a**	**4a**	**4a**
Input conditions		Load (%)	20	20	20	20	20	20	20	20
		T (^0^C)	25	25	25	25	25	25	25	25
		T (h)	24	24	24	24	16	24	24	5
Heuristic	Data	*ee*_R_ (%)	Observed, Predicted, and Residual values							
–	OD	Observed	93	0	26	65	11	67	52	54
H_1_	OD	Predicted	66.0	29.7	64.1	25.1	− 90.5	74.9	61.8	42.4
		Residual	27.0	− 29.7	− 38.1	39.9	101.5	− 7.9	− 9.8	11.6
	ODMC	Predicted	91.1	0.3	64.1	25.1	12.1	66.6	50.8	52.1
		Residual	1.9	− 0.3	− 38.1	39.9	− 1.1	0.4	1.2	1.9
H_2_	OD	Predicted	− 36.2	22.0	− 50.8	− 50.9	− 121.3	− 36.1	− 49.2	− 57.9
		Residual	129.2	− 22.0	76.8	115.9	132.3	103.1	101.2	111.9
	ODMC	Predicted	− 34.3	21.6	− 49.6	− 49.2	− 119.3	− 34.5	− 47.7	− 56.2
		Residual	127.3	− 21.6	75.6	114.2	130.3	101.5	99.7	110.2

^a^*OD* Original Data, *MC* Monte Carlo, *ODMC* OD + MC enriched dataset. *Nuc* = Nucleophile, *Cat* = Catalyst, *Load (%)* Catalyst loading (%), the symbols **3a** - **4d** are *Nuc* and *Cat*, see scheme 4

**Scheme 5.**
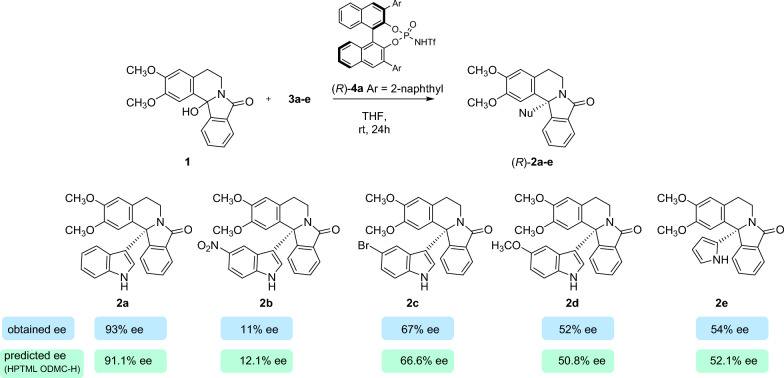
Experimentally obtained ee values *vs.* predicted (HPTML ODMC-H) for the obtention of **2a-e** using catalyst **4a**

The other HPTML models have notably larger residuals values, confirming our decision to discard them as good predictors for this type of reaction. In general, the best results are obtained with the HPTML ODMC H_1_ model. For a total of 6 out of 8 reactions the model almost perfectly predicts the observed values of *ee*_R_(%)_qrobs_ with residual values in the range *ee*_R_(%)_qrres_ = − 1.1–1.9% (reactions 1, 2, 5–8) (Table 9). The experimental and predicted values for the obtention of **2a-e** using catalyst **4a** are represented in Scheme 5. For the other two reactions, the model correctly predicts the absolute stereochemistry of the final products, although with a relatively higher error. In addition to the results of training and validations series, these results validate the HPTML ODMC H_1_ model as a useful predictor for enantioselective intermolecular α-amidoalkylation reactions. The Microsoft Excel software was used to run all these calculations. However, this HPTML calculation algorithm is slow because it is not automatic and need more than one software applications (MCDCalc, Excel) to run. Furthermore, the model is not available for use by other groups and requires some degree of expertise in Cheminformatics, so we decided to implement it on a public web server.

### MATEO web server

**Fig. 5:**
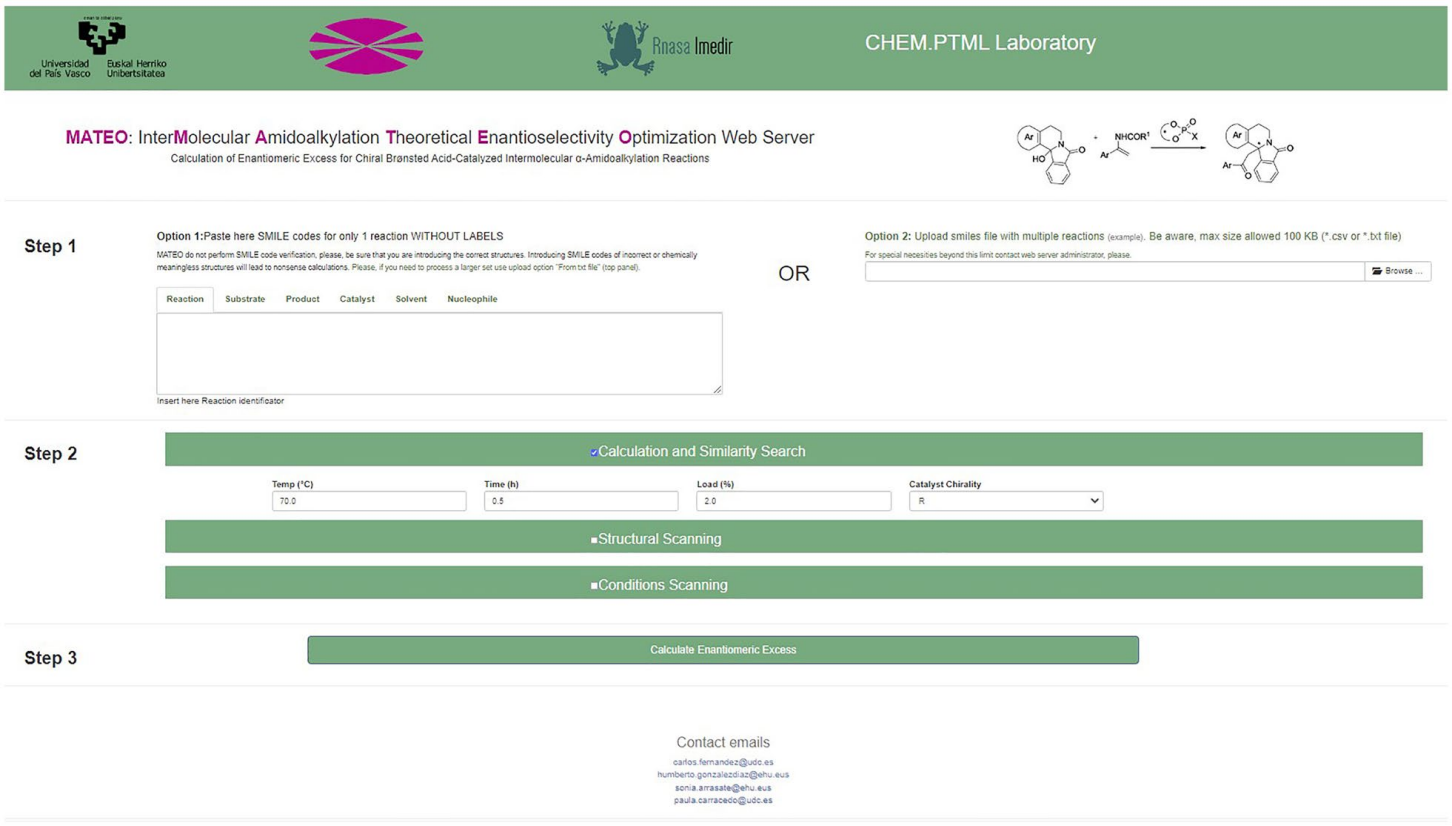
MATEO web server user interface

The HPTML model was implemented on a new public web server called MATEO: interMolecular Amidoalkylation Theoretical Enantioselectivity Optimization. MATEO server is available for public use online (free of charge) through the link: https://cptmltool.rnasa-imedir.com/CPTMLTools-Web/mateo. The graphical interface of the web server is shownin Fig. 5.Users worldwide can upload their own sets of query reactions to predict the values of *ee*_R_(%)_qrcalc_ under different experimental conditions (solvent, time, temperature, catalyst loading), see Table 10.

**Table 10:** MATEO Web server operational conditions

Stat.^a^	MATEO application operational conditions^b^		
	T (^o^C)	T (h)	Load (%)
Default	25.00	0.5	2.00
Min	− 78.00	0.5	2.00
Max	70.00	72.0	5.00
Step	20	1.0	1.00

^a^*Stat.* Statistical parameters for the input parameters (operational conditions) of all the reactions present in our dataset: *Max.* maximum value, *Min.* minimum value, *Step* minimal change allowed in one experimental condition

^b^Operational conditions: *T(*^*o*^*C)* temperature, *t(h)* reaction time, *Load(%)* catalyst loading

**Fig. 6:**
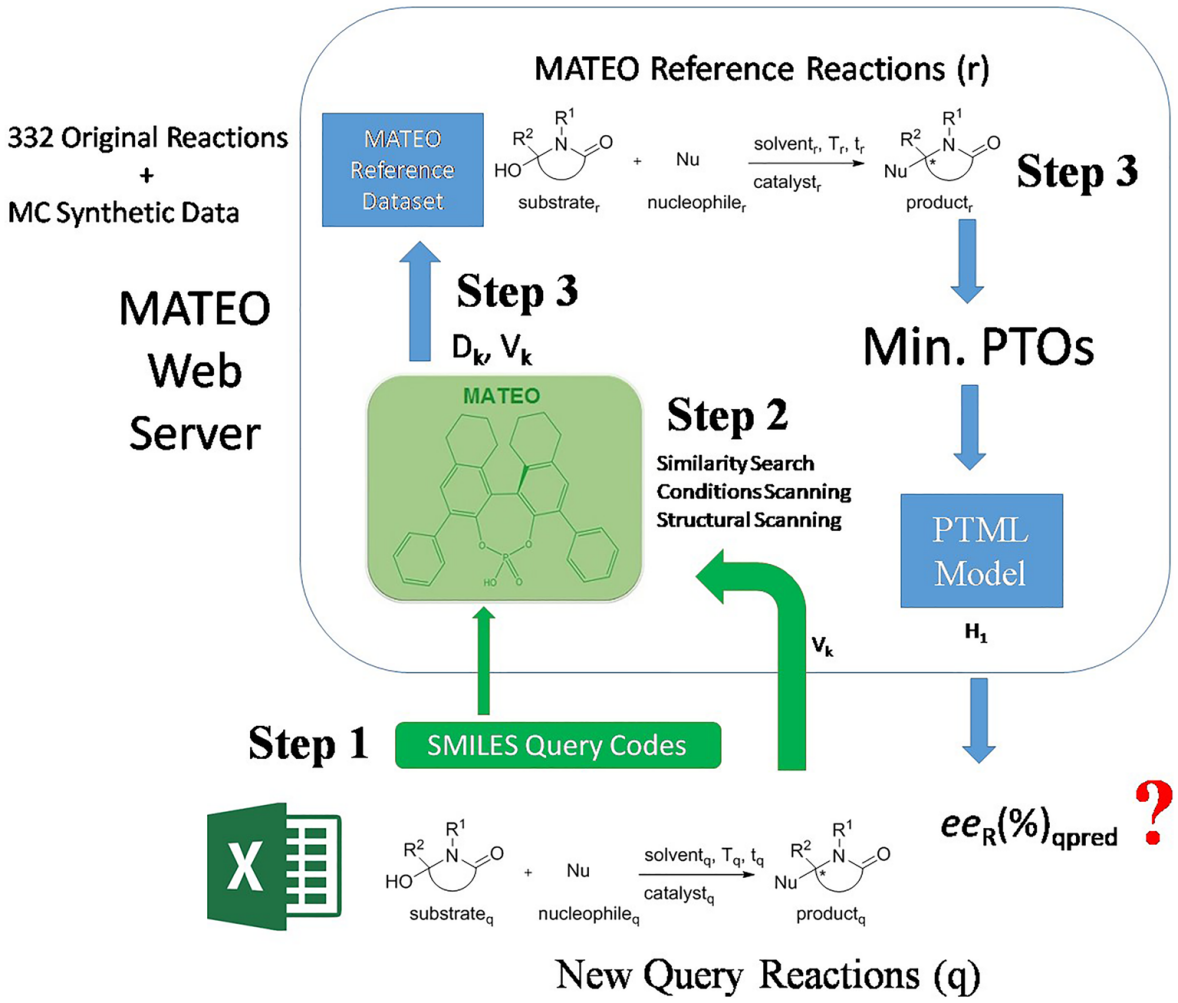
MATEO server use workflow

Figure 6 graphically illustrates (from bottom to top) the steps required to use this web server. Step 1 is to upload the chemical structures of all the molecules involved in the reaction. The server is required to upload the structures in the Simplified Molecular Input Line Entry Specification (SMILES) code format [65]. SMILES has become a simplified and memory-optimal way of managing molecular structures widely used in Cheminformatics today [66, 67]. These codes can be pasted directly on the web interface or uploaded as a text file. The server allows uploading large collections of reactions with different combinations of substrate, nucleophile, and catalyst. This could be useful for exploring large libraries of molecules (products, substrates, and nucleophiles) and/or for the design of new catalysts. The server also allows uploading of the solvent structure, making it easy to explore a large variety of solvents. In Step 2, three general types of calculations can be selected: (1) Similarity Search, (2) Structural Scan, or (3) Conditions Scan. Option (1) allows us to predict the enantiomeric excess values, in addition to obtaining a report of the most similar reactions from the references in our dataset. Option (2) allows uploading the specific structures (substrate, nucleophile, catalyst, and/or solvent) and running a scan of these molecules under reaction conditions similar to those reported in the literature. Option (3) allows to keep the structure parameters constant (same molecules), while the software performs a scan of different combinations of input variables (temperature, time, catalyst loading). Table 10 shows the range (minimum, maximum) and step of the variables allowed by the server.

In this context, Goodman et al. have recently developed a rule-based web tool BINOPtimal for the online selection of CPA catalysts in a related reaction, the addition of nucleophiles to imines, by analyzing the reagent structures [68]. MATEO is web server allows the user to make quantitative predictions of enantiomeric excess parameter *ee*_R_(%) at different reaction temperature, time, catalysts loading or solvent polarity, which are known factors that affect the enantioselectivity of α-amidoalkylation reactions. Therefore, MATEO web server will be useful to guide not only the catalyst selection but also the experimental conditions.

## Conclusions

In conclusion, we have shown that classic linear ML models are not very accurate in predicting the enantioselectivity of α-amidoalkylation reactions using physicochemical properties calculated with a Markov chain approach as input. Besides, these linear ML models do not allow detecting the most similar reaction directly from the model. The PTML algorithm outperforms the classic linear ML model using the same dataset and molecular descriptors. Moreover, the HPTML algorithm based on PTML model + heuristic rule allows direct detection of the most similar reference reactions. In addition, MC synthetic data re-sampling/enrichment procedures reduce the procedural error. The final HPTML model responds very well in computational experiments with validation series. The HPTML model also reproduces very well the experimental values of a new series of reactions studied experimentally by the first time in this work. Finally, the implementation of the HPTML model on the MATEO online server makes the algorithm available for public use worldwide with a user-friendly interface.

### Supplementary Information

**Additional file1: **The following files are available free of charge. General experimental methods; Synthetic procedures and structural determination for **2a-d**; Copies of HPLC chromatograms of racemic and enantioenriched **2a-d**; Copies of ^1^H and ^13^C NMR spectra

**Additional file2: **Dataset of reactions, molecular descriptors, SMILE codes, *etc*.

**Additional file3: **MATEO server reactions of reference

## Data Availability

MATEO web server was implemented for public use by experimental organic chemists, see link: https://cptmltool.rnasa-imedir.com/CPTMLTools-Web/mateo.The code of the software was uploaded to a GitHub repository and is available free for use by cheminformatics researchers with MIT license. The links are the following. For the MATEO server code the link is: https://github.com/glezdiazh/MATEO. For libraries used to calculate the molecular descriptors the link is: https://github.com/muntisa/RMarkovTI.All data files (SI00, SI01, and SI02) have been uploaded to a public data repository and are available for use free of charge under universal commons creative license (CC0). The links are, SI00.pdf file link: https://doi.org/10.6084/m9.figshare.21981740.v2, Additional file 2: https://doi.org/10.6084/m9.figshare.21971690.v2, and Additional file 3: https://doi.org/10.6084/m9.figshare.21971696.v2.

## Abbreviations

AI: Artificial intelligence
ANN: Artificial neural networks
CPA: Chiral phosphoric acid
GLR: General linear regression
ML: Machine learning
HPTML: Heuristic perturbation-theory and machine learning
LNN: Linear neural network
MARCH-INSIDE: Markov chain invariants for networks simulation and design
MATEO: InterMolecular amidoalkylation theoretical enantioselectivity optimization
MC: Monte carlo
ML: Machine learning
MLR: Multivariate linear regression
THF: Tetrahydrofuran
OD: Original data
PT: Perturbation theory
PTO: Perturbation theory operator
SE: Standard error
SEE: Standard error estimates
SMILE: Simplified molecular input line entry specification
*ee*_*R*_(%)_obs_: Observed enantiomeric excess (experimental) using (*R*)-Catalyst
*ee*_*R*_(%)_ref_: Enantiomeric excess of reference (experimental) using (*R*)-Catalyst
*ee*_*R*_(%)_calc_: Enantiomeric excess using (*R*)-Catalyst calculated using one reference
*ee*_*R*_(%)_pred_: Enantiomeric excess using (*R*)-Catalyst predicted by the model
*ee*_*R*_(%)_res_: Residual enantiomeric excess using (*R*)-Catalyst
