# Supplementary material for: MATEO: intermolecular α-amidoalkylation theoretical enantioselectivity optimization. Online tool for selection and design of chiral catalysts and products
Source: J Cheminform. 2024 Jan 23;16:9. doi: 10.1186/s13321-024-00802-7 (PMC10804835; doi:10.1186/s13321-024-00802-7)
Supplement: Supplementary file 1 — Additional file1: The following files are available free of charge. General experimental methods; Synthetic procedures and structural determination for 2a-d; Copies of HPLC chromatograms of racemic and enantioenriched 2a-d; Copies of 1H and 13C NMR spectra [file 13321_2024_802_MOESM1_ESM.pdf]

Table of Contents:

1. General experimental methods S2

2. Synthesis of racemic **2e** S2

3. Synthesis of enantioenriched isoindolo[1,2-*a*]isoquinolines **2a-d** S3

4. HPLC chromatograms of racemic and selected enantioenriched **2a-e**. S5

5. Copies of ^{1}H and ^{13}C NMR spectra S8

6. General reaction equations for the reference reactions in the database................. S13

7. Molecule codes S17

1. General experimental methods.

Melting points were determined in unsealed capillary tubes. IR spectra were obtained in film over NaCl pellets. NMR spectra were recorded at 20-25 ˚C, at 300 MHz for ^{1}H and 75.5 MHz for ^{13}C in $CDCl_3$ solutions, unless otherwise stated. Assignments of individual ^{13}C and ^{1}H resonances are supported by DEPT experiments and 2D correlation experiments (COSY, HSQCed or HMBC). Mass spectra were recorded under under chemical ionization (CI) at 230 eV. Exact mass was obtained using a TOF detector. TLC was carried out with 0.2 mm thick silica gel plates. Visualization was accomplished by UV light. Flash column chromatography was performed on silica gel (230-400 mesh) or on alumina (70-230 mesh). Chiral stationary phase HPLC was performed using a Chiralcel OD column (0.46 cm × 25 cm). Optical rotation was measured at 20 ºC with a sodium lamp at 589 nm and a path length of 1 dm. All solvents used in reactions were anhydrous and purified according to standard procedures. All air- or moisture-sensitive reactions were performed under argon; the glassware was dried (130 ˚C) and purged with argon. Racemic samples of **2a-d** were prepared according the procedure previously described.[1] A racemic sample of **2e** was prepared by the same procedure (see below). Phosphoramides **4a-d** were prepared according to literature procedures.[2] Phosphoric acid **5e** was used from commercial source (Aldrich, 98%).

2. Synthesis of racemic 2,3-dimethoxy-12b-(1*H*-pyrrol-2-yl)-5,12b-dihydroisoindolo[1,2-*a*]isoquinolin-8(6*H*)-one (2e).

$TiCl_4$ (0.14 mL, 1.4 mmol) was added dropwise to a solution of 12b-hydroxy-isoindoloisoquinolone **1**[3] (160 mg, 0.52 mmol) and pyrrole **3e** (0.03 mL, 0.52 mmol) in dry CH_2Cl_2 (10 mL) at –78 ºC. After 2 h stirring, NH_4Cl (saturated aqueous solution, 2 mL) was added, and the reaction mixture was allowed to warm up to room temperature. The organic layer was separated and the aqueous phase was extracted with CH_2Cl_2 (3× 15 mL). The combined organic extracts were dried (Na_2SO_4) and concentrated *in vacuo*. The

---

[1] Aranzamendi, E.; Sotomayor, N.; Lete, E. Bronsted acid catalyzed enantioselective alpha-amidoalkylation in the synthesis of isoindoloisoquinolines. *J. Org. Chem.* **2012**, 77, 2986-91.

[2] Nakashima, D.; Yamamoto, H. Design of chiral *N*-triflyl phosphoramide as a strong chiral Bronsted acid and its application to asymmetric Diels-Alder reaction. *J. Am. Chem. Soc.* **2006**, *128*, 9626-7.

[3] Collado, M. I.; Manteca, I.; Sotomayor, N.; Villa, M. J.; Lete, E. Metalation *vs* Nucleophilic Addition in the Reactions of *N*-Phenethylimides with Organolithium Reagents. Ready Access to Isoquinoline Derivatives *viaN*-Acyliminium Ions and Parham-Type Cyclizations. *J. Org. Chem*. **1997**, *62*, 2080-92.

crude mixture was purified by column chromatography (silica gel, hexane/ethyl acetate, 6:4) to obtain isoindoloisoquinoline **2e** (138 mg, 76%).

3. Synthesis of enantioenriched isoindolo[1,2-*a*]isoquinolines 2a-d.

***(R)*-12b-(1*H*-indol-3-yl)-2,3-dimethoxy-5,6-dihydroisoindolo[1,2-*a*]isoquinolin-8(12b*H*)-one (2a) (Table 8, entry 1)**. According to the Typical Procedure, **1** (62 mg, 0.20 mmol) was treated with indole **3a** (24 mg, 0.20 mmol) and **4a** (29.2 mg, 0.04 mmol, 20 mol %) in dry THF (5 mL) for 24h at rt, to afford isoindolo[1,2-*a*]isoquinoline **2a** (71 mg, 90 %), whose spectroscopic data are coincidental to those reported.[1] The enantiomeric excess was determined by HPLC to be 93% [Chiralcel OD, 15 % hexane : 2-propanol, 1 mL/min, t_r (*S*)= 26.32 min (3.48 %), t_r (*R*)= 30.23 min (96.52 %)]. ^{1}H NMR (300 MHz, $CDCl_3$): δ 2.72-2.77 (m, 1H), 3.14-3.22 (m, 2H), 3.85 (s, 3H), 3.88 (s, 3H), 4.37-4.41 (m, 1H), 6.40 (d, J = 8.1 Hz, 1H), 6.67 (s, 1H), 6.75 (d, J = 2.6 Hz, 1H), 6.79 (t, J = 7.6 Hz, 1H), 7.08 (t, J = 7.3 Hz, 1H), 7.25 (s, 1H), 7.31 (d, J = 8.2 Hz, 1H), 7.51-7.54 (m, 2H), 7.64-7.67 (m, 1H), 7.97-8.01 (m, 1H), 8.31 (broad s, 1H) ppm; ^{13}C{^{1}H} NMR (75.5 MHz, $CDCl_3$): δ 29.7, 34.9, 55.9, 56.2, 66.1, 110.9, 111.2, 111.8, 117.7, 119.8, 120.0, 122.4, 123.9, 124.0, 124.9, 126.5, 127.3, 128.6, 129.5, 131.88, 132.0, 136.9, 147.0, 148.5, 149.8, 167.2 ppm.

(*R*)-2,3-dimethoxy-12b-(5-nitro-1*H*-indol-3-yl)-5,6-dihydroisoindolo[1,2-*a*]isoquinolin-8(12b*H*)-one (2b) (Table 8, entry 5). According to the Typical Procedure, **1** (62 mg, 0.20 mmol) was treated with indole **3b** (32 mg, 0.20 mmol) and **4a** (29.2 mg, 0.04 mmol, 20 mol %) in dry THF (5 mL) for 24h at rt to afford isoindolo[1,2-*a*]isoquinoline **2b** (83 mg, 94%), whose spectroscopic data are coincidental to those reported.[1] The enantiomeric excess was determined by HPLC to be 11% [Chiralcel OD, 15 % hexane : 2-propanol, 1 mL/min, t_r (*S*)= 44.46 min (44.37 %), t_r (*R*)= 57.18 min (55.63 %)]. ^{1}H NMR (300 MHz, $CDCl_3$): δ 2.76-2.78 (m, 1H), 3.13-3.22 (m, 2H), 3.85 (s, 3H), 3.89 (s, 3H), 4.43-4.46 (m, 1H), 6.59 (s, 1H), 6.93 (d, J = 2.0 Hz, 1H), 7.22 (s, 1H), 7.25 (d, J = 2.0 Hz, 1H), 7.35 (d, J = 9 Hz, 1H), 7.56-7.65 (m, 2H), 7.71-7.72 (m, 1H), 8.01 (d, J = 9.0 Hz, 1H,), 8.10-8.04 (m, 1H), 8.9 (broad s, 1H) ppm; ^{13}C{^{1}H} NMR (75.5 MHz, DMSO-d_6): δ 28.6, 34.9, 55.9, 56.4, 65.6, 111.7, 112.7, 113.0, 115.8, 117.2, 118.9, 123.5, 124.1, 125.3, 127.1, 128.8, 129.5, 131.4, 131.7, 133.1, 140.7, 140.8, 147.5, 148.8, 150.0, 166.5 ppm.

(*R*)-12b-(5-bromo-1*H*-indol-3-yl)-2,3-dimethoxy-5,6-dihydroisoindolo[1,2-*a*]isoquinolin-8(12b*H*)-one (2c) (Table 8, entry 6). According to the Typical Procedure, **1** (62 mg, 0.2 mmol) was treated with indole **3c** (39 mg, 0.2 mmol) and **4a** (29.2 mg, 0.04 mmol, 20 mol %) in dry THF (5 mL) for 24h at rt to afford isoindolo[1,2-*a*]isoquinoline **2b** (94 mg, quant.%), whose spectroscopic data are coincidental to those reported.[1] The enantiomeric excess was determined by HPLC to be 67% [Chiralcel OD, 15 % hexane : 2-propanol, 1 mL/min, t_r (*S*)= 24.80 min (18.42 %), t_r (*R*)= 32.92 min (83.58 %)]. ^{1}H NMR (300 MHz, $CDCl_3$): δ 2.70-2.90 (m, 1H), 3.09-3.24 (m, 2H), 3.84 (s, 3H), 3.87 (s, 3H), 4.34-4.43 (m, 1H), 6.45 (s, 1H), 6.66 (s, 1H), 6.77 (d, *J* = 2.5 Hz, 1H), 7.14-7.22 (m, 3H), 7.54-7.57 (m, 2H), 7.62-7.65 (m, 1H), 7.98-8.01 (m, 1H), 8.49 (broad s, 1H) ppm; ^{13}C{^{1}H} NMR (75.5 MHz, $CDCl_3$): δ 28.6, 34.9, 55.97, 56.2, 65.8, 111.6, 111.8, 112.8, 113.4, 117.5, 122.3, 123.9, 124.0, 125.4, 126.5, 127.3, 127.7, 128.9, 129.2, 131,8, 132.1, 135.7, 147.1, 148.6, 149.4, 167.3 ppm.

(*R*)-2,3-dimethoxy-12b-(5-methoxy-1*H*-indol-3-yl)-5,6-dihydroisoindolo[1,2-*a*]isoquinolin-8(12b*H*)-one (2d) (Table 8, entry 7). According to the Typical Procedure, **1** (62 mg, 0.2 mmol) was treated with indole **3d** (29 mg, 0.2 mmol) and **4a** (29.2 mg, 0.04 mmol, 20 mol %) in dry THF (5 mL) for 24h at rt to afford isoindolo[1,2-*a*]isoquinoline **2b** (85 mg, quant.%), whose spectroscopic data are coincidental to those reported.[1] The enantiomeric excess was determined by HPLC to be 52% [Chiralcel OD, 15 % hexane : 2-propanol, 1 mL/min, t_r (*S*)= 25.34 min (23.82 %), t_r (*R*)= 34.11 min (76.18 %)]. ^{1}H NMR (300 MHz, $CDCl_3$): δ 2.73-2.82 (m, 1H), 3.09-3.28 (m, 2H), 3.41 (s, 3H), 3.84 (s, 3H), 3.87 (s, 3H), 4.38-4.44 (m, 1H), 5.71-5.72 (m, 1H), 6.57 (s, 1H), 6.71-6.74 (m, 2H), 7.18 (d, *J* = 8.8 Hz, 1H), 7.26 (s, 1H), 7.50-7.59 (m, 2H), 7.69-7.71 (m, 1H), 7.98-8.01 (m, 1H), 8.32 (broad s, 1H) ppm; ^{13}C{^{1}H} NMR (75.5 MHz, $CDCl_3$): δ 28.9, 35.8, 55.2, 55.9, 56.2, 66.3, 101.1, 110.9, 111.7, 112.1, 112.7, 117.0, 123.7, 124.3, 125.3, 127.2, 127.3, 128.6, 129.5, 131.9, 132.0, 132.2, 147.1, 148.5, 149.9, 153.8, 167.1 ppm.

4. HPLC chromatograms of racemic and selected enantioenriched 2a-e

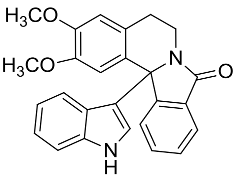

2a, racemic mixture

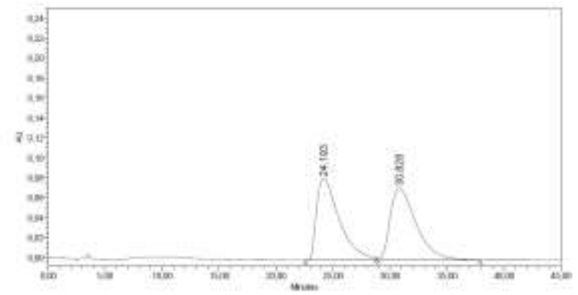

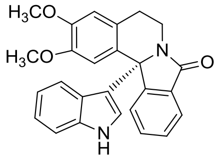

(*R*)-2a, 93 % *ee*,

Table 8, entry 1

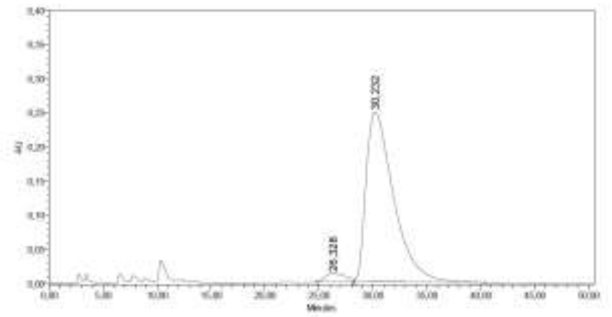

	RT	Area	% Area	Height
1	26,328	1592410	3,48	12232
2	30,232	44149254	96,52	248815

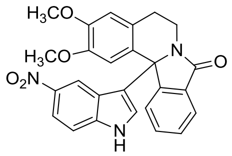

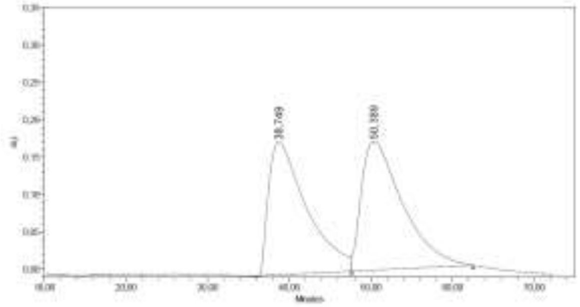

2b, racemic mixture

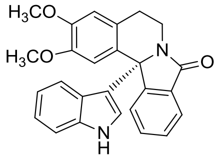

(*R*)-2b, 11 % *ee*,

Table 8, entry 5

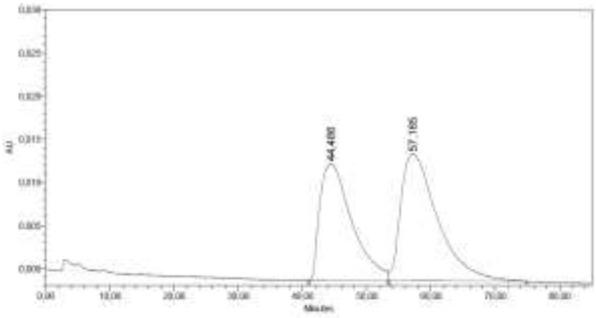

	RT	Area	% Area	Height
1	44,486	4691623	44,37	13552
2	57,185	5881437	55,63	14787

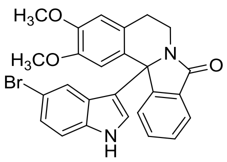

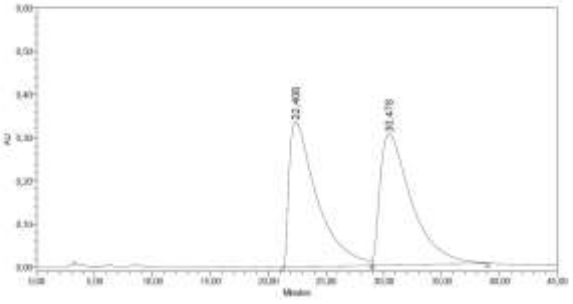

2c, racemic mixture

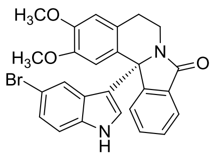

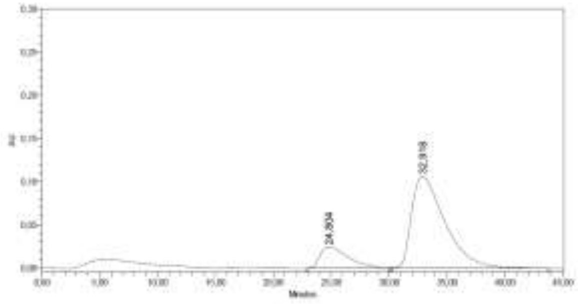

***(R)*-2c**, 67% *ee*,

Table 8, entry 6

	RT	Area	% Area	Height
1	24,604	4237253	16,42	25181
2	32,918	21574792	83,58	107005

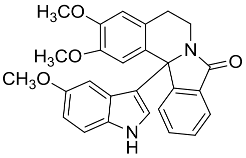

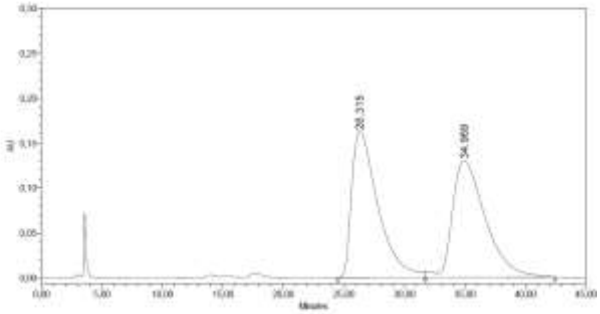

2d, racemic mixture

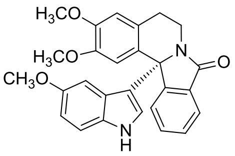

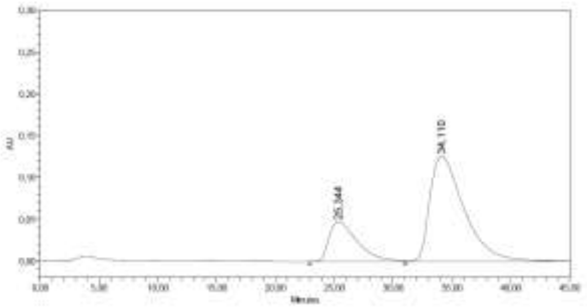

***(R)*-2d**, 52 % *ee*,

Table 8, entry 7

	RT	Area	% Area	Height
1	25,344	6043197	23,82	47902
2	34,110	25723430	76,18	126569

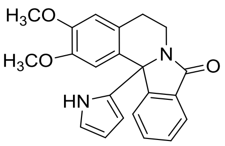

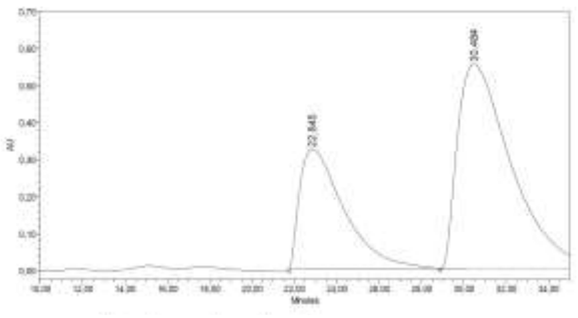

2e, racemic mixture

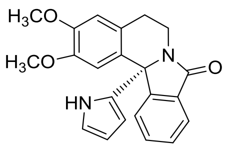

(*R*)-2e, 54 % *ee*,

Table 8, entry 7

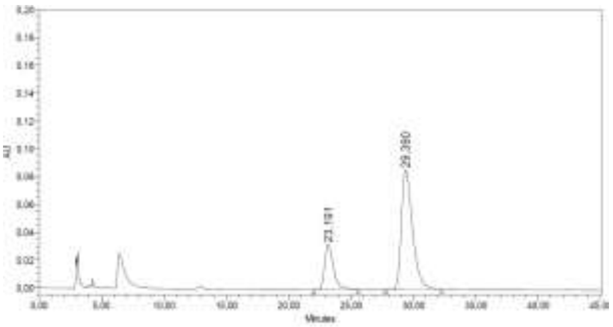

	RT	Area	% Area	Height
1	23.191	1569318	22.87	32404
2	29.390	5293577	77.13	85889

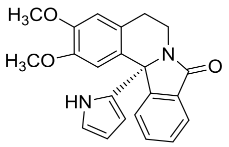

(*R*)-2e, 24 % *ee*,

Table 8, entry 15

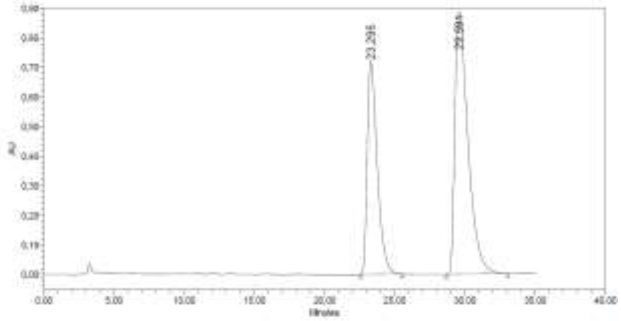

	RT	Area	% Area	Height
1	23.295	35583127	38.17	718953
2	29.501	57658288	61.83	887291

5. Copies of ^{1}H and ^{13}C NMR of 2a-e

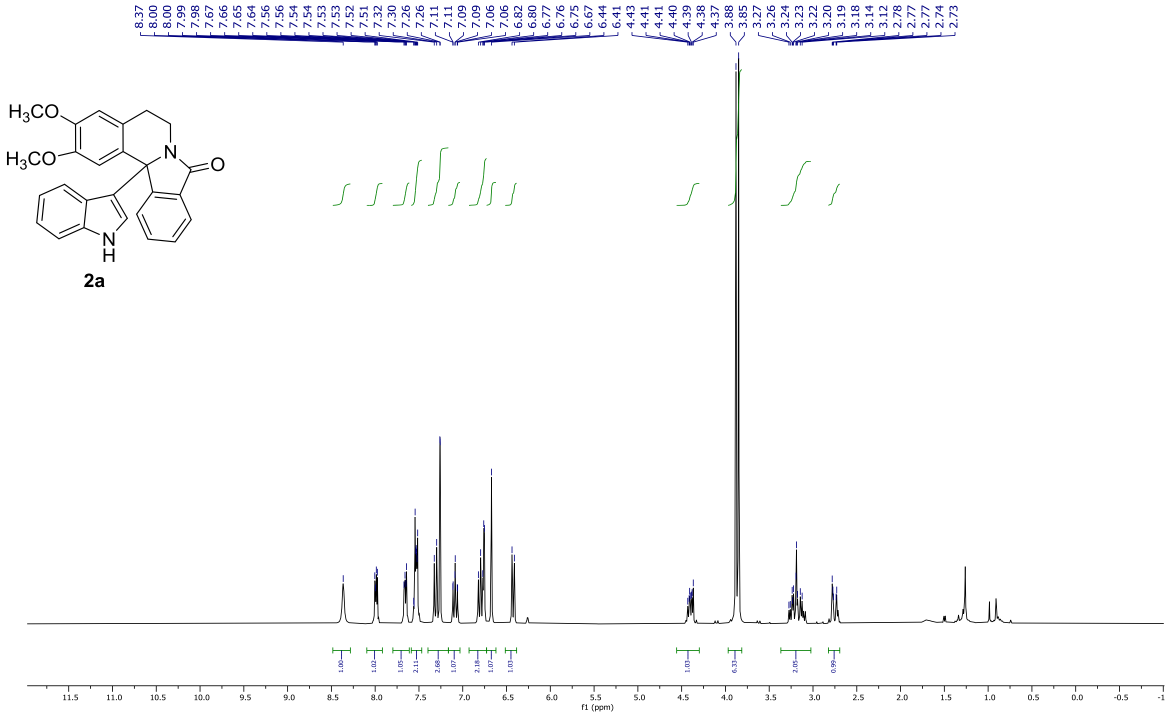

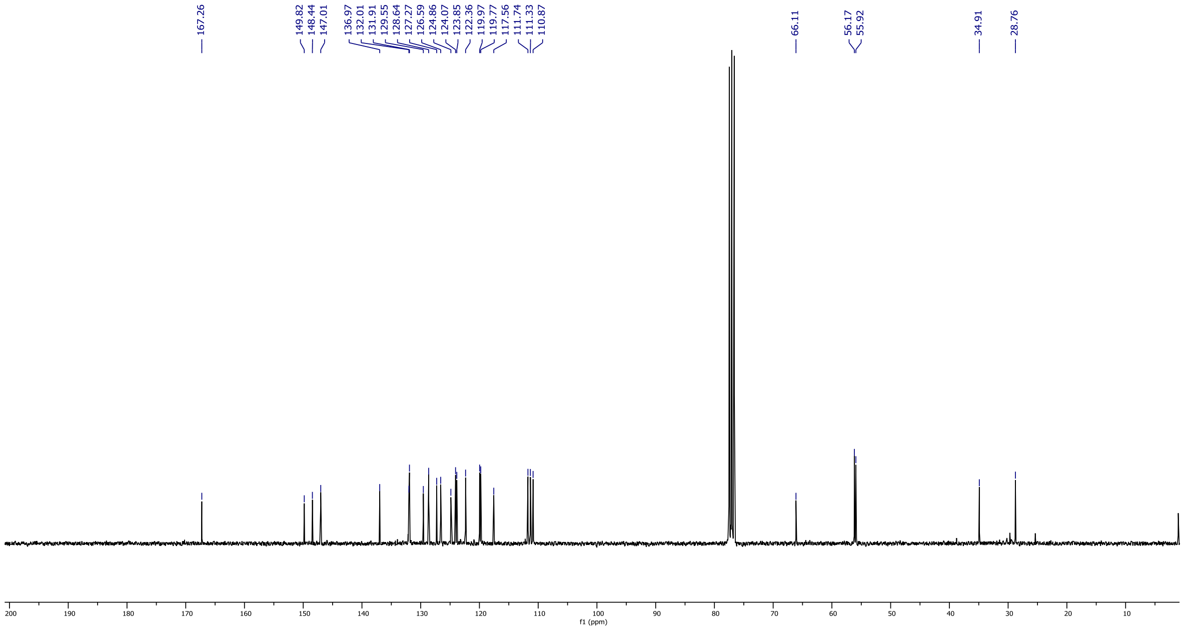

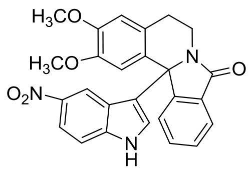
H3CO
H3CO
O2N
N
O
N
H

2b

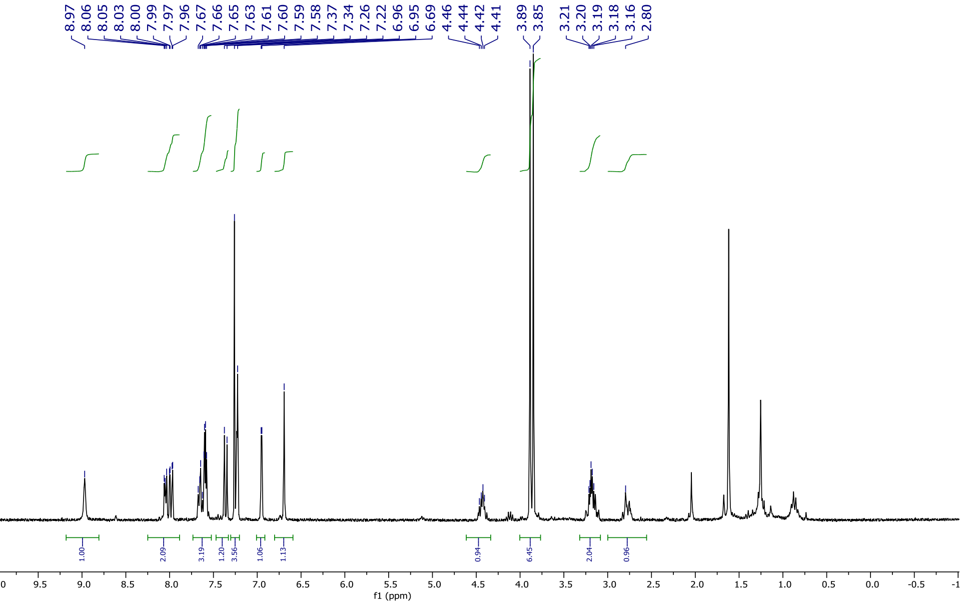
8.97
8.06
8.05
8.03
8.00
7.99
7.97
7.96
7.67
7.66
7.65
7.63
7.61
7.60
7.59
7.58
7.37
7.34
7.26
7.22
6.96
6.95
6.69
4.46
4.44
4.42
4.41
3.89
3.85
3.21
3.20
3.19
3.18
3.16
2.80
1.00
2.09
3.19
1.20
3.56
1.06
1.13
0.94
6.45
2.04
0.96
f1 (ppm)

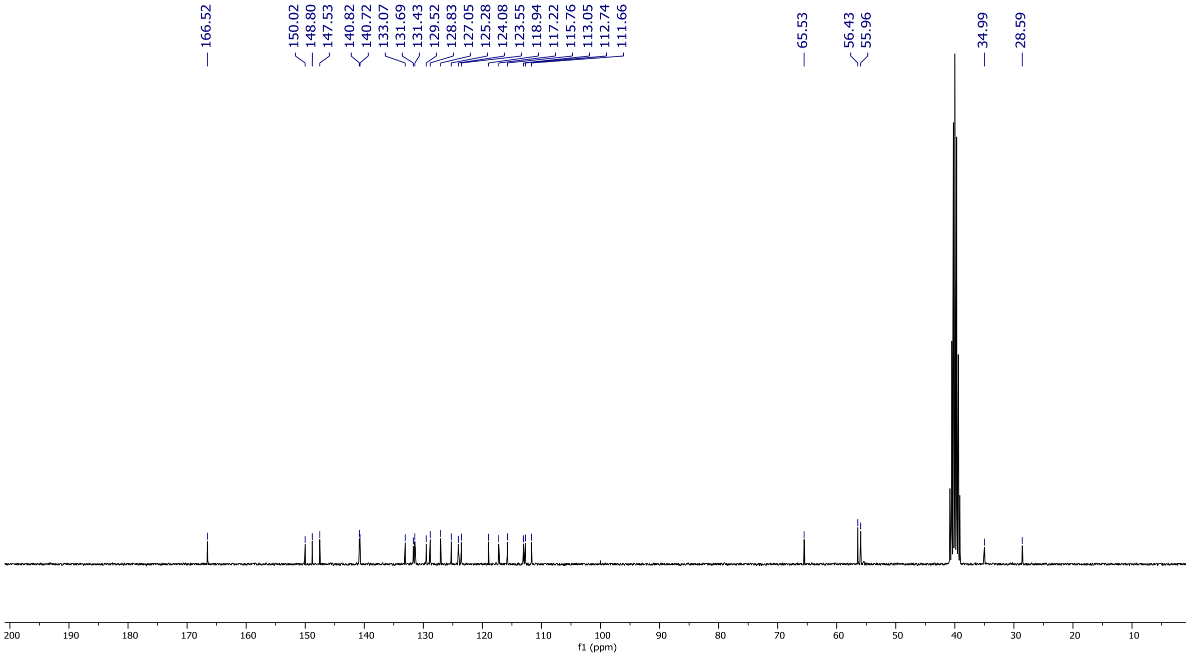
166.52
150.02
148.80
147.53
140.82
140.72
133.07
131.69
131.43
129.52
128.83
127.05
125.28
124.08
123.55
118.94
117.22
115.76
113.05
112.74
111.66
65.53
56.43
55.96
34.99
28.59
f1 (ppm)

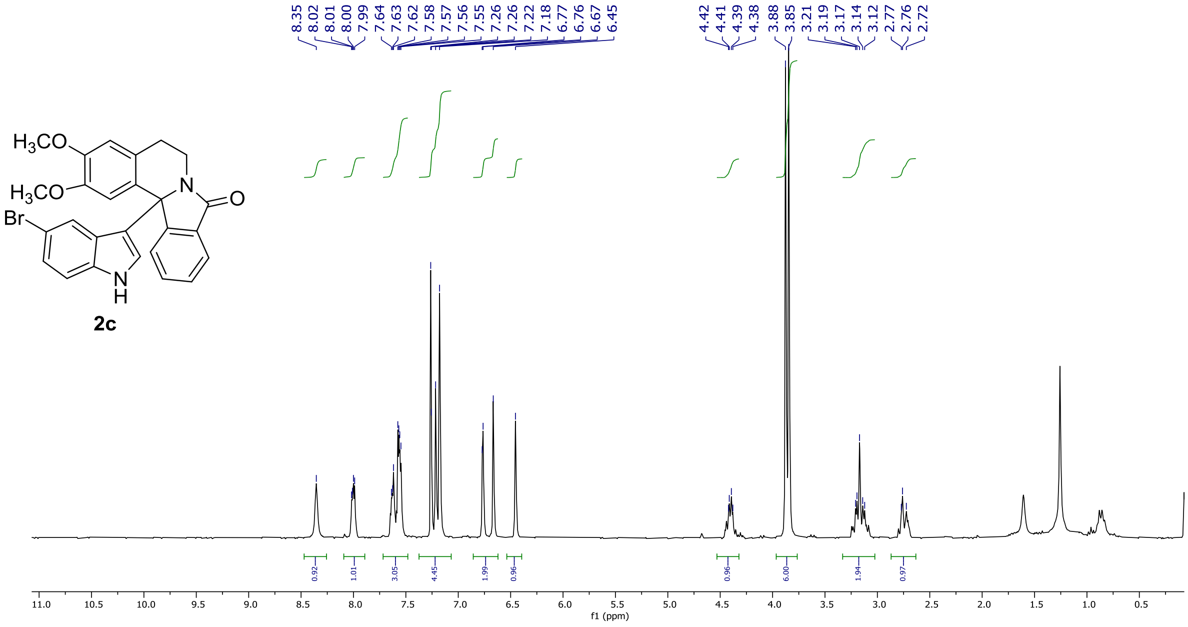
H3CO
H3CO
Br
N
O
NH
2c
8.35 8.02 8.01 8.00 7.99 7.64 7.63 7.62 7.58 7.57 7.56 7.55 7.26 7.26 7.22 7.18 6.77 6.76 6.67 6.45
4.42 4.41 4.39 4.38 3.88 3.85 3.21 3.19 3.17 3.14 3.12 2.77 2.76 2.72
0.92 1.01 3.05 4.45 1.99 0.96 0.96 6.00 1.94 0.97
11.0 10.5 10.0 9.5 9.0 8.5 8.0 7.5 7.0 6.5 6.0 5.5 5.0 4.5 4.0 3.5 3.0 2.5 2.0 1.5 1.0 0.5
f1 (ppm)

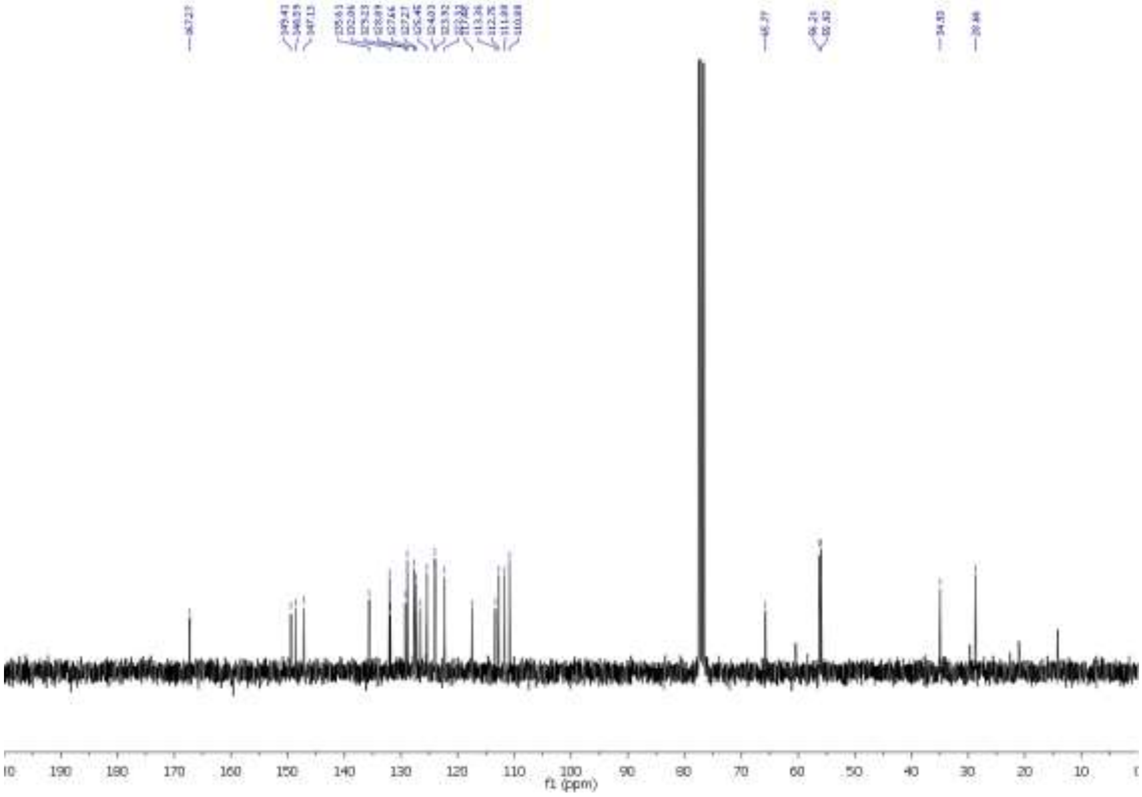
190 180 170 160 150 140 130 120 110 100 90 80 70 60 50 40 30 20 10
f1 (ppm)

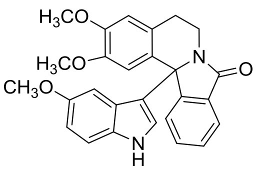

H_3CO
H_3CO
CH_3O
N
O
N
H

2d

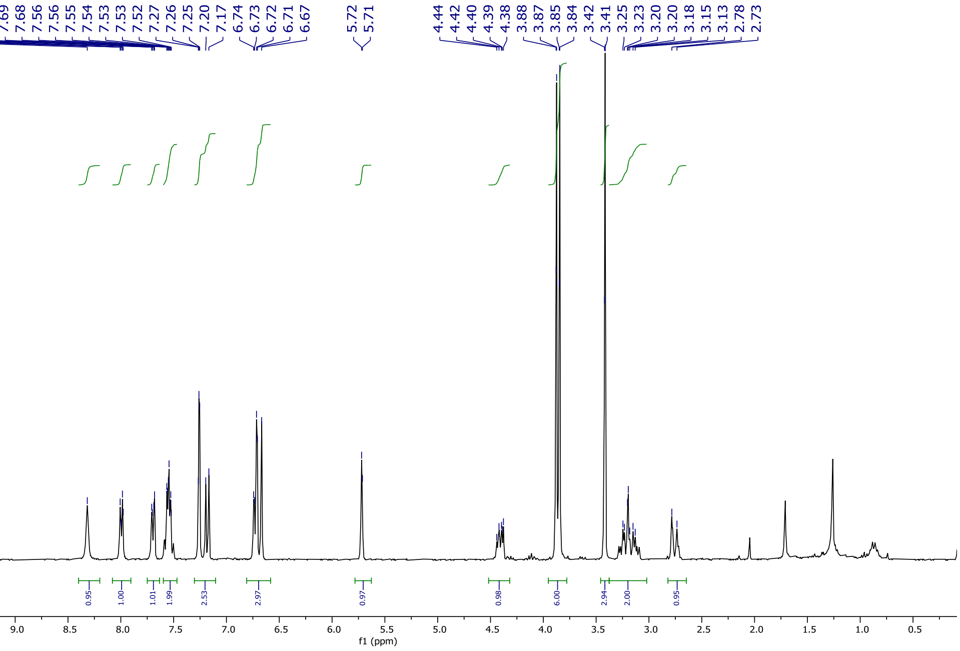

8.32 8.01 8.00 7.98 7.98 7.71 7.70 7.69 7.68 7.56 7.56 7.55 7.54 7.53 7.53 7.52 7.27 7.26 7.25 7.20 7.17 6.74 6.73 6.72 6.71 6.67
5.72 5.71
4.44 4.42 4.40 4.39 4.38 3.88 3.87 3.85 3.84 3.42 3.41 3.25 3.23 3.20 3.20 3.18 3.15 3.13 2.78 2.73
0.95 1.00 1.01 1.99 2.53 2.97 0.97 0.98 6.00 2.94 2.00 0.95
11.0 10.5 10.0 9.5 9.0 8.5 8.0 7.5 7.0 6.5 6.0 5.5 5.0 4.5 4.0 3.5 3.0 2.5 2.0 1.5 1.0 0.5
f1 (ppm)

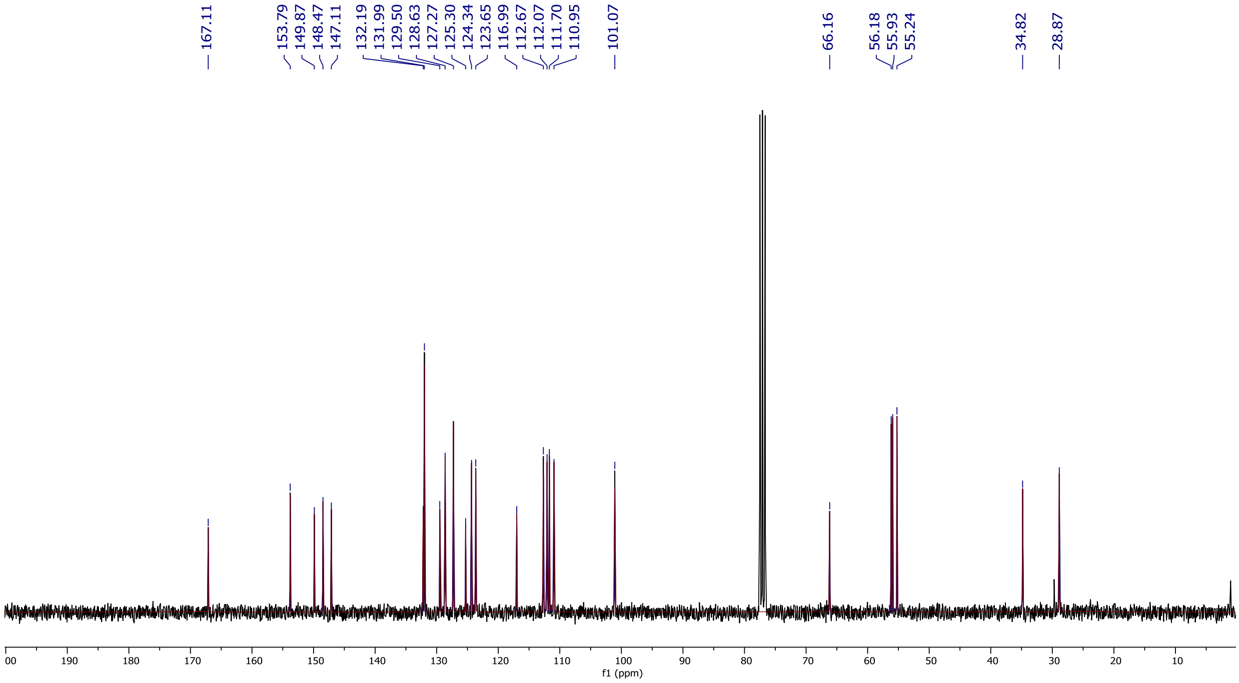

167.11
153.79 149.87 148.47 147.11
132.19 131.99 129.50 128.63 127.27 125.30 124.34 123.65 116.99 112.67 112.07 111.70 110.95
101.07
66.16
56.18 55.93 55.24
34.82
28.87
00 190 180 170 160 150 140 130 120 110 100 90 80 70 60 50 40 30 20 10
f1 (ppm)

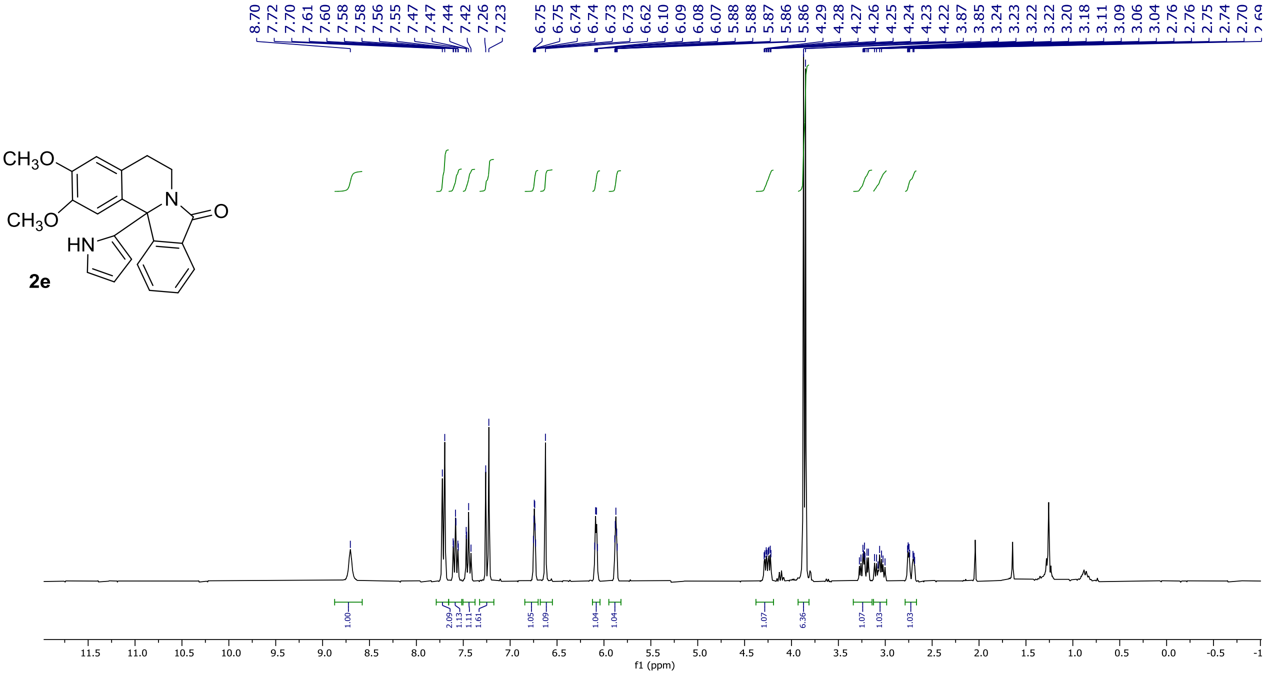

CH3O
CH3O
HN
N
O

2e

8.70
7.72
7.70
7.61
7.60
7.58
7.56
7.55
7.47
7.44
7.42
7.26
7.23
6.75
6.75
6.74
6.74
6.73
6.73
6.62
6.10
6.09
6.08
6.07
5.88
5.88
5.87
5.86
4.29
4.28
4.27
4.26
4.25
4.24
4.23
4.22
3.87
3.85
3.24
3.23
3.22
3.22
3.20
3.18
3.11
3.09
3.06
3.04
2.76
2.76
2.75
2.74
2.70
1.00
2.09
1.13
1.11
1.61
1.05
1.09
1.04
1.04
1.07
6.36
1.07
1.03
1.03
f1 (ppm)

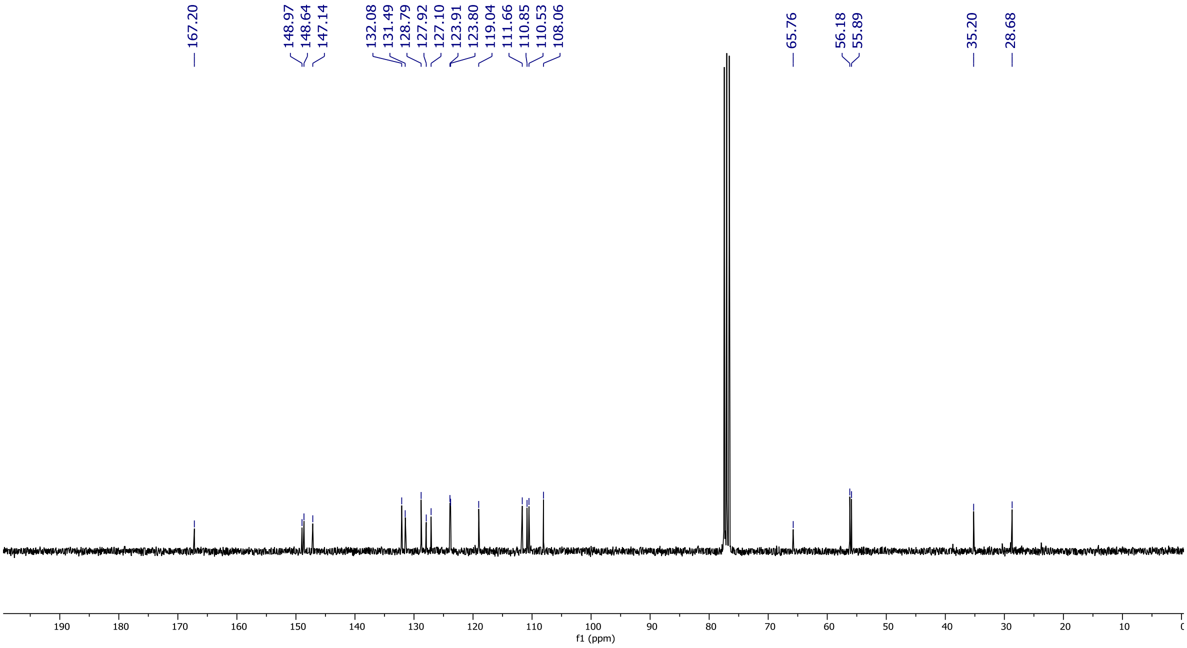

167.20
148.97
148.64
147.14
132.08
131.49
128.79
127.92
127.10
123.91
123.80
119.04
111.66
110.85
110.53
108.06
65.76
56.18
55.89
35.20
28.68
f1 (ppm)

6. General reaction equations for the reference reactions in the database

In the following schemes the general equations for the reactions included in the database are shown including the bibliographic reference. FileSI02.xls contains the full experimental detail for each reaction (substrate, nucleophile, catalyst, reaction conditions, yield, and ee). Molecule codes are depicted in the next section.

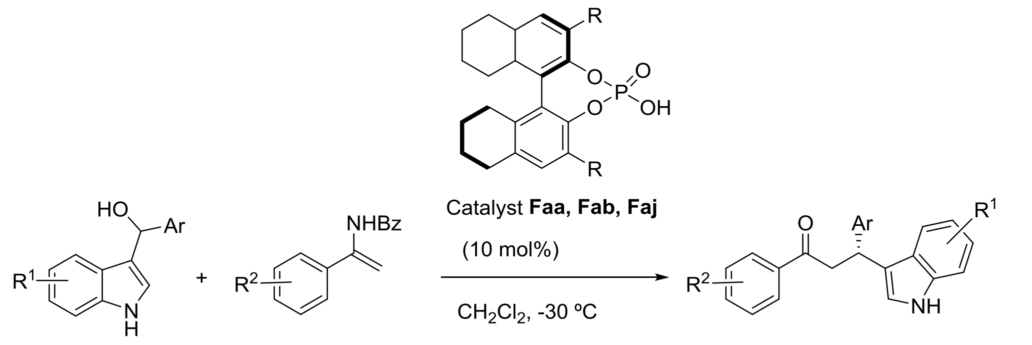

Substrates **S05** Nucleophiles **Nuf** Products **P05**

38 reactions in the database, up to 96%ee

Scheme S1. General reaction from: Guo, Q. X.; Peng, Y. G.; Zhang, J. W:; Song, L.; Feng, Z.; Gong, L. Z. *Org. Lett.* **2009**, 11, 4620;

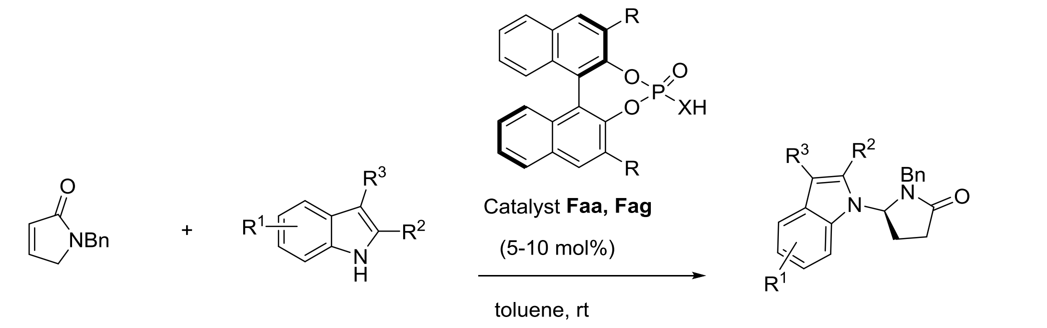

Substrate **S02he** Nucleophiles **Nua** Products **P02**

38 reactions in the database, up to 95%ee

Scheme S2. General reaction from: Xie, Y.; Zhao, Y.; Qian, B.; Yang, L.; Xia, C.; Huang, H. *Angew. Chem. Int. Ed.* **2011**, *50*, 5682;

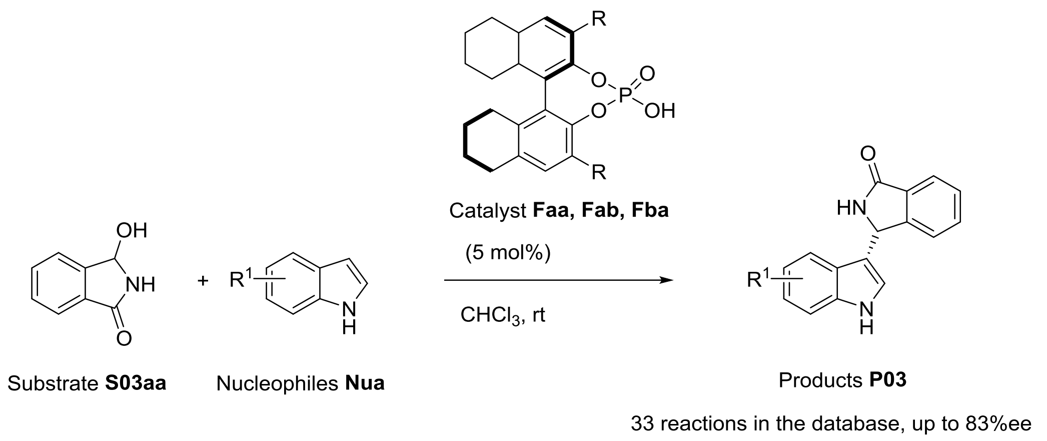

Scheme S3. General reaction from: Yu, X.; Lu, A.; Wang, Y.; Wu, G.; Song, H.; Zhou, Z.; Tang, C. *Eur. J. Org. Chem.* **2011**, 892;

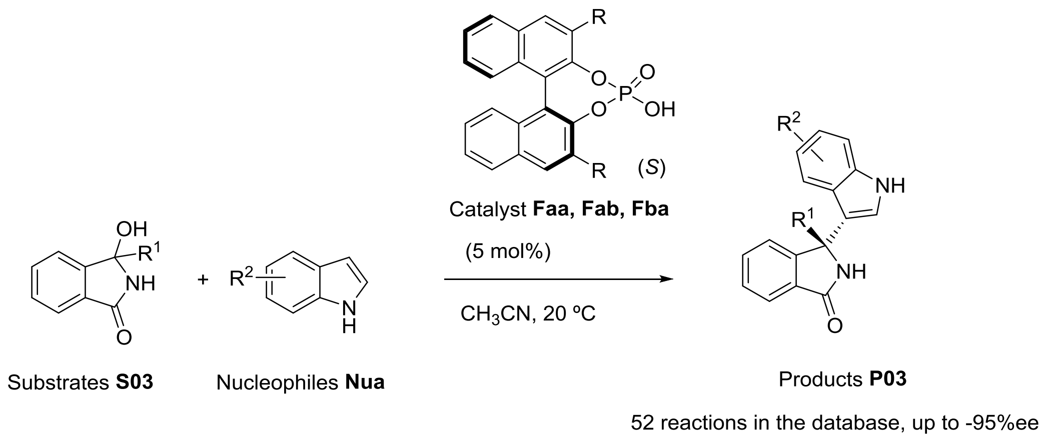

Scheme S4. General reaction from: Yu, X.; Lu, A.; Wang, Y.; Wu, G.; Song, H.; Zhou, Z.; Tang, C. *Eur. J. Org. Chem.* **2011**, 3060;

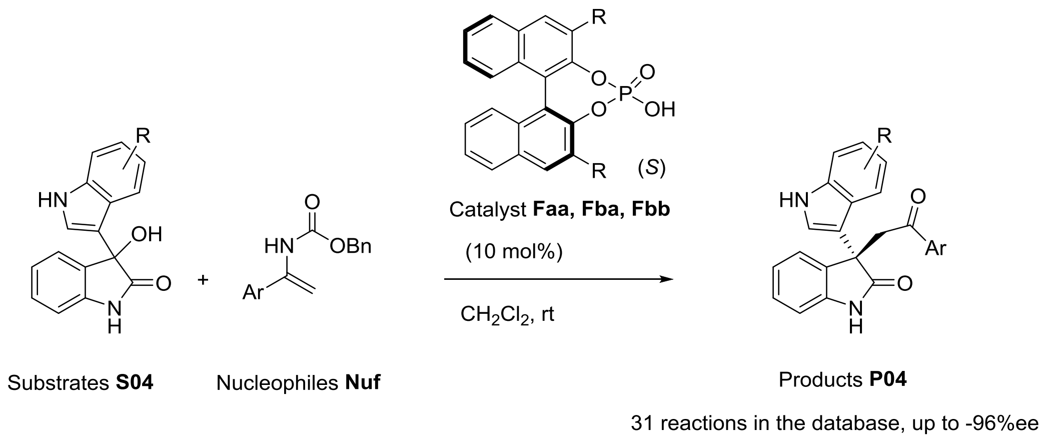

Scheme S5. General reaction from: Guo, C.; Song, J.; Huang, J. Z.; Chen, P. H.; Luo, S. W.; Gong, L. Z. *Angew. Chem. Int. Ed.* **2012**, *51*, 1046.

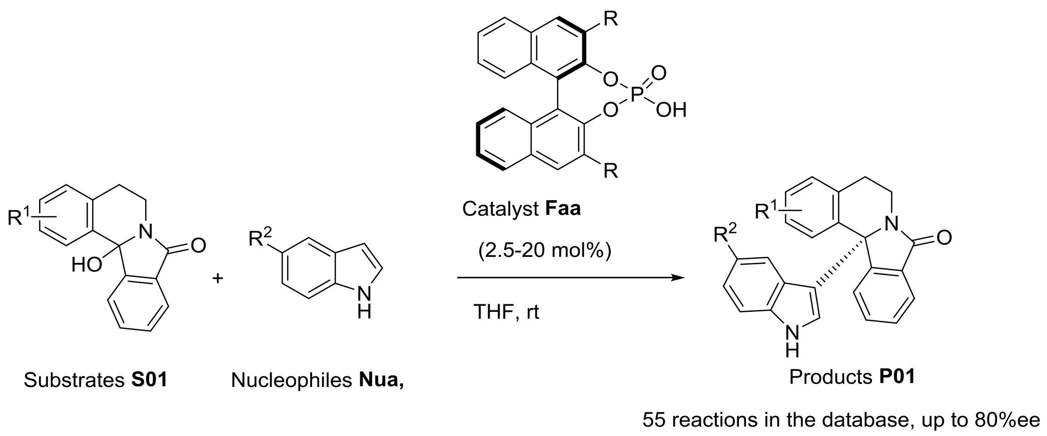

Scheme S6. General reaction from: Aranzamendi, E.; Sotomayor, N.; Lete, E. *J. Org. Chem.* **2012**, *77*, 2986.

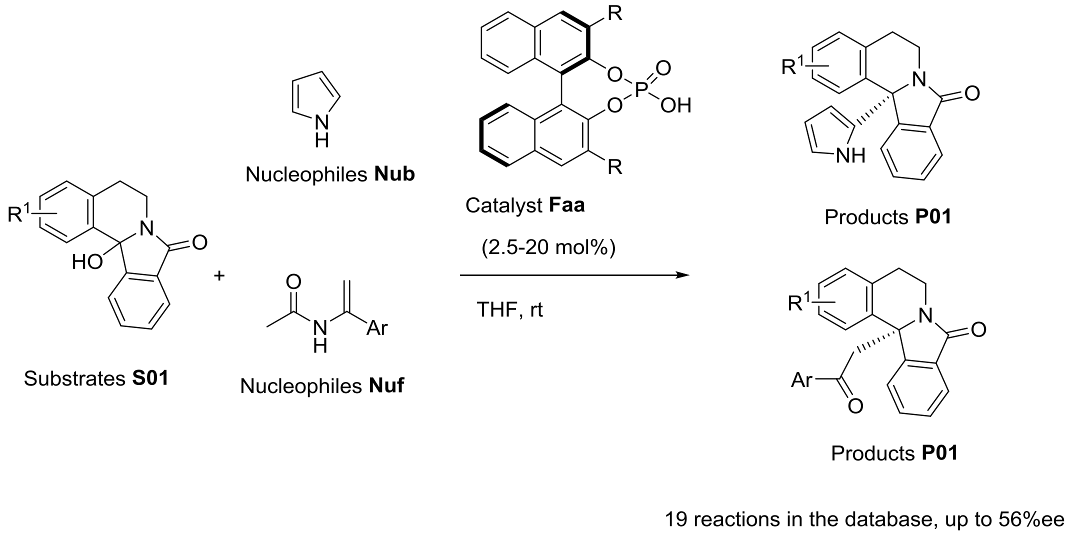

Scheme S7. General reaction from: E. Aranzamendi, Doctoral Thesis, 2014, UPV/EHU (unpublished results), and Aranzamendi, E.; Arrasate, S. Sotomayor, N.; González-Díaz, H.; Lete, E. *ChemistryOpen.* **2016**, *557*, 540.

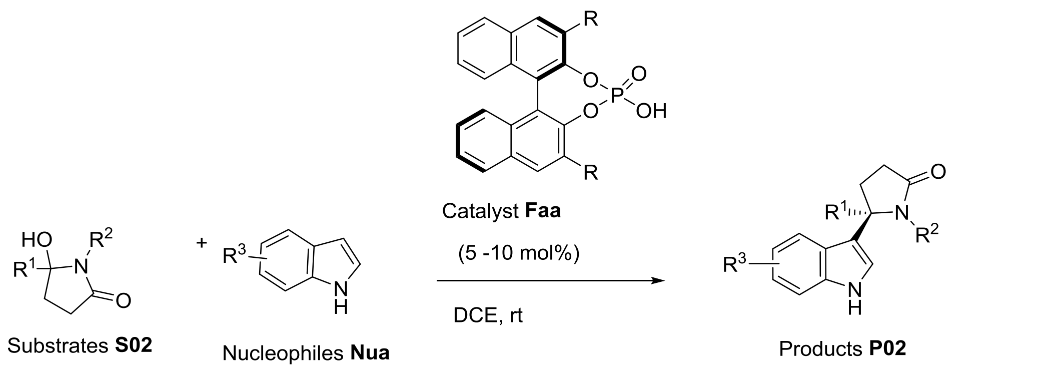

29 reactions in the database, up to 99%ee

Scheme S8. General reaction from: Courant, T.; Kumarn, S.; He, L.; Retailleau, P.; Masson, G. *Adv. Synth. Catal.* **2013**, *355*, 836;

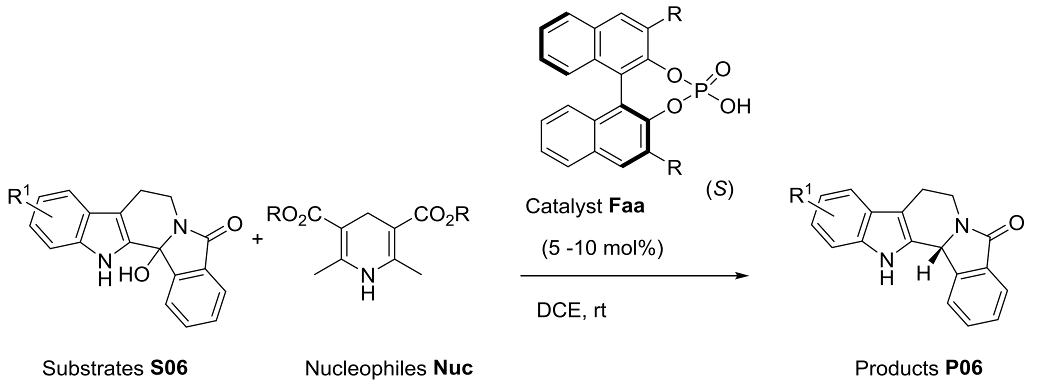

29 reactions in the database, up to -90%ee

Scheme S9. General reaction from: Yin, Q.; Wang, S. -G.; You, S. -L. *Org. Lett.* **2013**, *15*, 2688.

7. Molecule codes

SUBSTRATES

S01

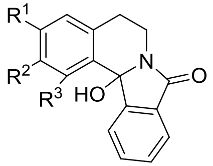

S01aa $R^1 = R^2 = OCH_3$, $R^3 = H$
S01ba $R^1 = R^3 = OCH_3$ $R^1 = H$
S01ca $R^1 = R^3 = R^1 = OCH_3$

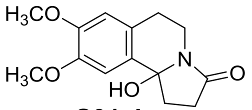

S01ab

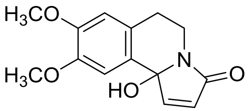

S01ac

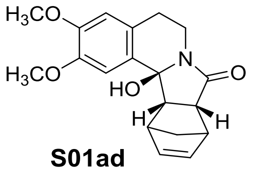

S01ad

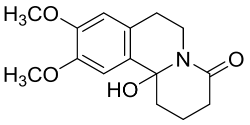

S01ae

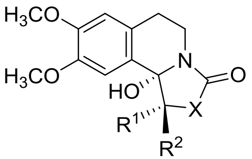

S01af X = NCH_3, $R^1 = R^2 = CH_3$
S01ag X = NCH_3, $R^1 = H$, $R^2 = Bn$
S01ah X = S, $R^1 = R^2 = H$
S01ai X = O, $R^1 = R^2 = H$
S01aj X = NCH_3, $R^1 = CH_3$, $R^2 = Bn$
S01ak X = S, $R^1 = H$, $R^2 = Bn$
S01al X = S, $R^1 = CH_3$, $R^2 = H$
S01am X = S, $R^1 = Ph$, $R^2 = CH_3$

S02

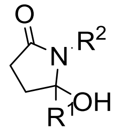

S02aa R^1 = H, R^2 = Ph
S02ac R^1 = H, R^2 = 4-BrC_6H_4
S02ad R^1 = H, R^2 = 4-IC_6H_4
S02ae R^1 = H, R^2 = Bn
S02aa R^1 = H, R^2 = Ph
S02ac R^1 = H, R^2 = 4-BrC_6H_4
S02ad R^1 = H, R^2 = 4-IC_6H_4

S02bb R^1 = CH_3, R^2 = 4-$CH_3C_6H_4$
S02be R^1 = CH_3, R^2 = Bn
S02bf R^1 = CH_3, R^2 = 2-$CH_3OC_6H_4CH_2$
S02bg R^1 = CH_3, R^2 = 4-$CH_3OC_6H_4CH_2$
S02bh R^1 = CH_3, R^2 = allyl

S02ce R^1 = Et, R^2 = Bn
S02de R^1 = propyl, R^2 = Bn
S02ee R^1 = *n*Bu, R^2 = Bn
S02fe R^1 = pentyl, R^2 = Bn
S02ge R^1 = Bn, R^2 = Bn

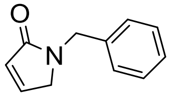

S02he

S03

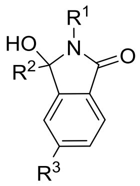

S03

S03aa R^1 = H, R^2 = H, R^3 = H
S03ab R^1 = H, R^2 = H, R^3 = Br
S03ba R^1 = H, R^2 = CH_3, R^3 = H
S03ca R^1 = H, R^2 = Et, R^3 = H
S03da R^1 = H, R^2 = Bu, R^3 = H

S03ea R^1 = H, R^2 = allyl, R^3 = H
S03fa R^1 = H, R^2 = Ph, R^3 = H
S03ac R^1 =CH_3, R^2 = R^3 = H
S03ad R^1 = Ph, R^2 = R^3 = H
S03da R^1 = H, R^2 = Bu, R^3 = H

S04

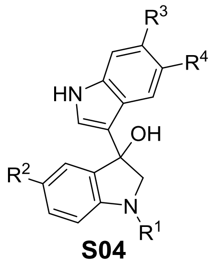

S04

S04aa R^1 = R^2 = R^3 = R^4 = H
S04ac R^1 = tertbutylacetyl, R^2 = R^3 = R^4 = H
S04ca R^1 = R^2 = R^3 = H, R^4 = Br
S04da R^1 = R^2 = R^3 = H, R^4 = OCH_3
S04ea R^1 = R^2 = R^3 = H, R^4 = Cl
S04fa R^1 = R^2 = H, R^3 = CH_3, R^4 = H
S04ga R^1 = R^2 = R^3 = H, R^4 = F

S04ha R^1 = R^2 = R^3 = F, R^4 = H
S04ad R^1 = H, R^2 = F, R^3 = R^4 = H
S04ae R^1 = H, R^2 = CH_3, R^3 = R^4 = H
S04fe R^1 = H, R^2 = R^3 = CH_3, R^4 = H
S04fd R^1 = H, R^2 = F, R^3 = CH_3, R^4 = H
S04hd R^1 = H, R^2 = F, R^3 = H, R^4 = F

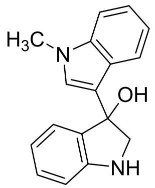

S04ba

S05

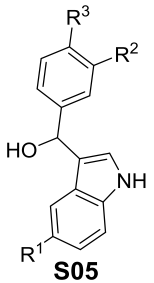

S05

S05aa $R^1 = R^2 = R^3 = H$
S05ab $R^1 = R^2 = H, R^3 = CH_3$
S05ac $R^1 = R^2 = H, R^3 = Cl$
S05ad $R^1 = R^2 = H, R^3 = Br$
S05ae $R^1 = R^2 = H, R^3 = CN$
S05af $R^1 = R^2 = H, R^3 = CF_3$
S05ag $R^1 = H, R^2 = F, R^3 = H$
S05ah $R^1 = H, R^2 = NO_2, R^3 = H$
S05ai $R^1 = H, R^2 = CN, R^3 = H$
S05aj $R^1 = H, R^2 = R^3 = F$
S05ak $R^1 = H, R^2 = Cl, R^3 = F$
S05be $R^1 = Cl, R^2 = H, R^3 = CN$
S05ce $R^1 = Br, R^2 = H, R^3 = CN$
S05de $R^1 = CH_3, R^2 = H, R^3 = CN$

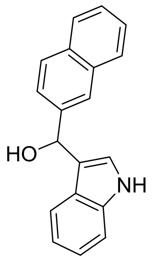

S05al

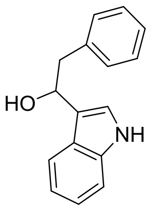

S05am

S06

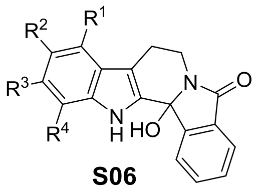

S06

S06aa $R^1 = R^2 = R^3 = R^4 = H$
S06ba $R^1 = CH_3, R^2 = R^3 = R^4 = H$
S06ca $R^1 = H, R^2 = CH_3, R^3 = R^4 = H$
S06da $R^1 = R^2 = H, R^3 = CH_3, R^4 = H$
S06ea $R^1 = R^2 = R^3 = H, R^4 = CH_3$
S06fa $R^1 = H, R^2 = OCH_3, R^3 = R^4 = H$
S06ga $R^1 = H, R^2 = Cl, R^3 = R^4 = H$
S06ha $R^1 = H, R^2 = Br, R^3 = R^4 = H$
S06ia $R^1 = R^2 = H, R^3 = Br, R^4 = H$
S06ja $R^1 = H, R^2 = F, R^3 = R^4 = H$

S07-S09

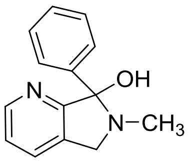

S07aa

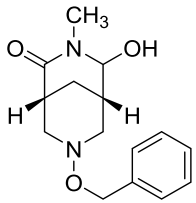

S08aa

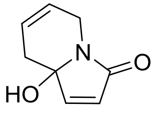

S09aa

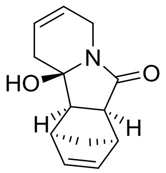

S09ab

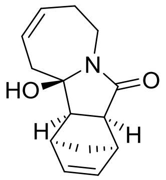

S09bb

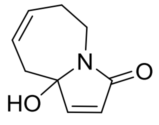

S09ba

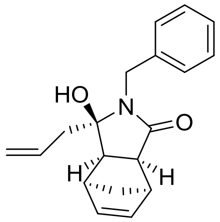

S09cc

NUCLEOPHILES

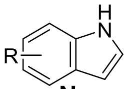

Nua

**Nua01**	R = H	**Nua08**	R = 5-NO_2	**Nua15**	R = 2,3-CH_3	**Nua22**	R = 7-OBn
**Nua02**	R = 6-OCH_3	**Nua09**	R = 6-F	**Nua16**	R =2-Et-3-CH_3	**Nua23**	R = 6-OBn
**Nua03**	R = 5-OCH_3	**Nua10**	R = 5-F	**Nua17**	R = 2-CH_3-5-pent	**Nua24**	R = 5-OBn
**Nua04**	R = 5-Br	**Nua11**	R =7-CH_3	**Nua18**	R = 2-BrEt	**Nua25**	R = 4-OBn
**Nua05**	R = 6-Cl	**Nua12**	R = 5-CH_3	**Nua19**	R = 2,3-CH_3-5-Cl		
**Nua06**	R = 6-Cl	**Nua13**	R = 3-CH_3	**Nua20**	R = 2-Ph		
**Nua07**	R = 5-Cl	**Nua14**	R= 2-CH_3	**Nua21**	R = 2-(2´-CH_3)C_6H_4		

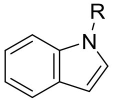

Nua26 R = Bn
Nua33 R = CH_3

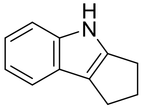

Nua27

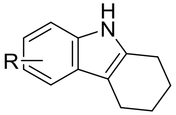

Nua28 R = H
Nua29 R = 6-CH_3
Mua30 R = 6-Cl

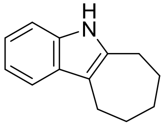

Nua31

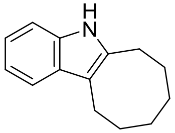

Nua32

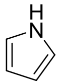

Nub01

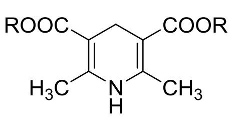

Nuc01 R = *t*Bu
Nuc02 R = Et

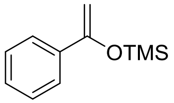

Nud01

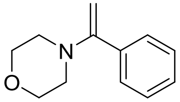

Nue01

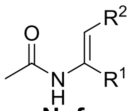

Nuf

**Nuf01**	R^1 = Ph, R^2 = H	**Nuf22**	R^1 =5,6,7,8-tetrahydronapht-2-yl R^2 = H	**Nuf97**	R^1 = 2-naphthyl, R^2 = CH_3
**Nuf110**	R^1 = 4-$CH_3C_6H_4$, R^2 = H	**Nuf23**	R^1 = Ph, R^2 = CH_3	**Nuf98**	R^1 =5,6,7,8-tetrahydronaphthyl, R^2 = CH_3
**Nuf12**	R^1 = 4-$OCH_3C_6H_4$, R^2 = H	**Nuf87**	R^1 = 4-CH_3, R^2 = CH_3	**Nuf101**	R^1 = 3,4-ClC_6H_4 , R^2 = H
**Nuf13**	R^1 = 4-$CH_3CONHC_6H_4$, R^2= H	**Nuf88**	R^1 = 4-$OCH_3C_6H_4$, R^2 = CH_3	**Nuf102**	R^1 = 4-CH_3OCOPh, R^2 = H
**Nuf14**	R^1 = 4-BrC_6H_4, R^2 = H	**Nuf89**	R^1 = 4-$CH_3CONHC_6H_4$, R^2 = CH_3	**Nuf103**	R^1 = 3-CNC_6H_4, R^2 = H
**Nuf15**	R^1 = 4-ClC_6H_4, R^2 = H	**Nuf90**	R^1 = 4-BrC_6H_4, R^2 = CH_3	**Nuf105**	R^1 = pyridine-3-yl, R^2 = H
**Nuf16**	R^1 = 4-FC_6H_4, R^2 = H	**Nuf91**	R^1 = 4-ClC_6H_4, R^2 = CH_3	**Nuf106**	R^1 = 3-(1´-(phenylsulfonyl)-1H-pyrrol-3yl, R^2 = H
**Nuf17**	R^1 = 4-$NO_2C_6H_4$, R^2 = H	**Nuf92**	R^1 = 4-FC_6H_4, R^2 = CH_3	**Nuf104**	R^1 = 2,3-dihydrobenzo[b][1,4]dioxin-6-yl R^2 = H
**Nuf18**	R^1 = 3-$OCH_3C_6H_4$, R^2 = H	**Nuf93**	R^1 = 4-$NO_2C_6H_4$, R^2 = CH_3	**Nuf107**	R^1 = tiophene-2-yl, R^2 = H
**Nuf19**	R^1 = 3,4-$(CH_3)_2C_6H_3$, R^2 = H	**Nuf94**	R^1 = 3-$OCH_3C_6H_4$, R^2 = CH_3	**Nuf108**	R^1 = benzofuran-2-yl, R^2 = H
**Nuf20**	R^1 =2,5-$(CH_3)_2C_6H_3$, R^2 = H	**Nuf95**	R^1 = 3,4-$(CH_3)_2C_6H_3$, R^2 = CH_3	**Nuf109**	R^1 = styryl,R^2 = H
**Nuf21**	R^1 = 2-naphthyl, R^2 = H	**Nuf96**	R^1 =2,5-$(CH_3)_2C_6H_3$, R^2 = CH_3		

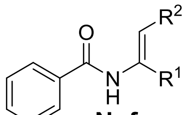

Nuf

**Nuf24**	R^1 = 4-$OCH_3C_6H_4$, R^2 = H	**Nuf29**	R^1 = 3,4-$CH_3C_6H_4$, R^2 = H	**Nuf36**	R^1 = Ph, R^2 = CH_3
**Nuf25**	R^1 = 4-$CH_3CONHC_6H_4$, R^2 =H	**Nuf30**	R^1 =2,5-$(CH_3)_2C_6H_3$, R^2 = H	**Nuf02**	R^1 = Ph, R^2 = H
**Nuf26**	R^1 = 4-BrC_6H_4, R^2 = H	**Nuf31**	R^1 = 2-naphthyl, R^2 = H	**Nuf03**	R^1 = 4-ClC_6H_4, R^2 = H
**Nuf27**	R^1 = 4-FC_6H_4, R^2 = H	**Nuf32**	R^1 = 3-$OCH_3C_6H_4$, R^2 = H	**Nuf04**	R^1 = 4-$CH_3C_6H_4$,R^2 =H
**Nuf28**	R^1 = 4-$NO_2C_6H_4$, R^2 = H	**Nuf33**	R^1 =5,6,7,8-tetrahydronaphthyl R^2 = H	**Nuf05**	R^1 = Ph, R^2 = CH_3

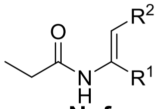

Nuf

**Nuf34**	R^1 = 4-$CH_3C_6H_4$, R^2 = H	**Nuf40**	R^1 = 4-FC_6H_4, R^2 = H	**Nuf45**	R^1 = 2-naphthyl, R^2 = H
**Nuf35**	R^1 = 4-$OCH_3C_6H_4$, R^2 = H	**Nuf41**	R^1 = 4-$NO_2C_6H_4$, R^2 = H	**Nuf46**	
**Nuf37**	R^1 = 4-ClC_6H_4, R^2 = H	**Nuf42**	R^1 = 3-$OCH_3C_6H_4$, R^2 =H		
**Nuf38**	R^1 = 4-$CH_3CONHC_6H_4$, R^2= H	**Nuf43**	R^1 = 3,4-$(CH_3)_2C_6H_3$, R^2= H	**Nuf47**	R^1 = Ph, R^2 = CH_3
**Nuf39**	R^1 = 4-BrC_6H_4, R^2 = H	**Nuf44**	R^1 =2,5-$(CH_3)_2C_6H_3$, R^2 = H	**Nuf06**	R^1 = Ph, R^2 = H

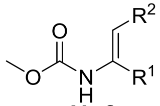

Nuf

**Nuf48**	R^1 = 4-ClC_6H_4, R^2 = H	**Nuf54**	R^1 = 4-FC_6H_4, R^2 = H	**Nuf59**	R^1 = 2-naphthyl, R^2 = H
**Nuf49**	R^1 = 4-$CH_3C_6H_4$, R^2 = H	**Nuf55**	R^1 = 4-$NO_2C_6H_4$, R^2 = H	**Nuf60**	
**Nuf50**	R^1 = 4-$OCH_3C_6H_4$, R^2 = H	**Nuf56**	R^1 = 3-$OCH_3C_6H_4$, R^2 =H		
**Nuf51**	R^1 = 4-$CH_3CONHC_6H_4$, R^2= H	**Nuf57**	R^1 = 3,4-$(CH_3)_2C_6H_3$, R^2= H	**Nuf61**	R^1 = Ph, R^2 = CH_3
**Nuf52**	R^1 = 4-BrC_6H_4, R^2 = H	**Nuf58**	R^1 =2,5-$(CH_3)_2C_6H_3$, R^2 = H	**Nuf07**	R^1 = Ph, R^2 = H

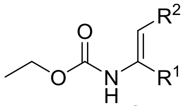

Nuf

**Nuf62**	R^1 = 4-ClC_6H_4, R^2 = H	**Nuf68**	R^1 = 4-FC_6H_4, R^2 = H	**Nuf73**	R^1 = 2-naphthyl, R^2 = H
**Nuf63**	R^1 = 4-$CH_3C_6H_4$, R^2 = H	**Nuf69**	R^1 = 4-$NO_2C_6H_4$, R^2 = H	**Nuf74**	
**Nuf64**	R^1 = 4-$OCH_3C_6H_4$, R^2 = H	**Nuf70**	R^1 = 3-$OCH_3C_6H_4$, R^2= H		
**Nuf65**	R^1 = 4-$CH_3CONHC_6H_4$, R^2= H	**Nuf71**	R^1 = 3,4-$(CH_3)_2C_6H_3$,R^2 = H	**Nuf75**	R^1 = Ph, R^2 = CH_3
**Nuf66**	R^1 = 4-BrC_6H_4, R^2 = H	**Nuf72**	R^1 =2,5-$(CH_3)_2C_6H_3$, R^2 = H	**Nuf08**	R^1 = Ph, R^2 = H

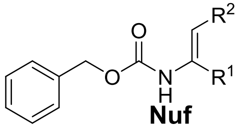

Nuf

**Nuf76**	R^1 = 4-$OCH_3C_6H_4$, R^2 = H	**Nuf81**	R^1 = 3-$OCH_3C_6H_4$, R^2= H	**Nuf86**	R^1 = Ph, R^2 = CH_3
**Nuf77**	R^1 = 4-$CH_3CONHC_6H_4$, R^2= H	**Nuf82**	R^1 = 3,4-$(CH_3)_2C_6H_4$, R^2=H	**Nuf09**	R^1 = Ph, R^2 = H
**Nuf78**	R^1 = 4-BrC_6H_4, R^2 = H	**Nuf83**	R^1 =2,5-$(CH_3)_2C_6H_4$, R^2 = H	**Nuf10**	R^1 = 4-$CH_3C_6H_4$, R^2 = H
**Nuf79**	R^1 = 4-FC_6H_4, R^2 = H	**Nuf84**	R^1 = 2-naphthyl, R^2 = H	**Nuf85**	
**Nuf80**	R^1 = 4-$NO_2C_6H_4$, R^2 = H	**Nuf11**	R^1 = 4-ClC_6H_4, R^2 = H		

CATALYSTS

Phosphoric acids

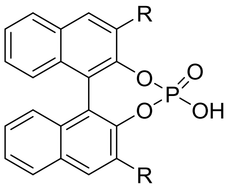

Fa (*R*) Fb *(S)*

**Faa01**	R = H	**Faa08**	R = 4-$CH_3OC_6H_4$	**Fba15**	R = $POPh_2$
**Fba01**	R = H	**Faa09**	R = 1-naphthyl	**Faa16**	R = 4-$CH_3C_6H_4$
**Faa02**	R = 9-anthracyl	**Fba09**	R = 1-naphthyl	**Faa17**	R = 3,5-$F_2C_6H_3$
**Fba02**	R = 9-anthracyl	**Faa10**	R = 2,4,6-(iPr)$_3C_6H_2$	**Faa18**	R = 4-(2´,4´,6´-$(CH_3)_3C_6H_2$)C_6H_5
**Faa03**	R = $SiCH_3Ph_2$	**Fba10**	R = 2,4,6-(iPr)$_3C_6H_2$	**Faa19**	R = 2,4,6-$(CH_3)_3C_6H_2$
**Faa04**	R = 9-phenanthryl	**Faa11**	R = $SiPh_3$	**Faa20**	R = 3,5-$(CH_3)_2C_6H_3$
**Faa05**	R = 2-naphthyl	**Fba11**	R = $SiPh_3$	**Faa21**	R = 4-(4´-tBuC_6H_4)C_6H_4
**Fba05**	R = 2-naphthyl	**Faa12**	R = 3,5-$(CF_3)_2C_6H_3$	**Faa22**	R = 4-(4´-tBu-2´,6´-$(CH_3)_2C_6H_3$)C_6H_4
**Faa06**	R = Ph	**Fba12**	R = 3,5-$(CF_3)_2C_6H_3$	**Faa23**	R = CF_3
**Fba06**	R = Ph	**Fba13**	R = 4-ClC_6H_4	**Faa24**	R = 3,5-(tBu)$_2C_6H_3$
**Faa07**	R = 4-$NO_2C_6H_4$	**Fba14**	R = 1,1`-biphenyl	**Faa25**	R = 4-tBuC_6H_5

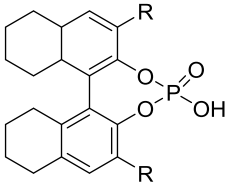

Fa (*R*) Fb (*S*)

**Fab01**	**R = 4-ClC_6H_4**	**Fab09**	**R = 4-$NO_2C_6H_4$**	**Fab18**	**R = 3,5-$(CH_3)_2C_6H_3$**
**Fab02**	**R = $SiPh_3$**	**Fab10**	**R = 4-$CH_3OC_6H_4$**	**Fab19**	**R = 4-(4´-tBuC_6H_4)C_6H_4**
**Fab03**	**R = Ph**	**Fab11**	**R = 1-naphthyl**	**Fab20**	**R = 4-(4´-tBu-2´,6´-$(CH_3)_2C_6H_3$)C_6H_4**
**Fab04**	**R = 2-naphthyl**	**Fab12**	**R = 2,4,6-(iPr)$_3C_6H_2$**	**Fab21**	**R = CF_3**
**Fab05**	**R = 9-phenanthryl**	**Fab13**	**R = 3,5-$(CF_3)_2C_6H_3$**	**Fab22**	**R = 3,5-(tBu)$_2C_6H_3$**
**Fbb05**	**R = 9-phenanthryl**	**Fab14**	**R = $POPh_2$**	**Fab23**	**R = $SiCH_3Ph_2$**
**Fab06**	**R = H**	**Fab15**	**R = 4-$CH_3C_6H_4$**	**Fab24**	**R = 3,5-F_2Ph**
**Fab07**	**R = 9-anthracyl**	**Fab16**	**R = 4-(2´,4´,6´-$(CH_3)_3C_6H_2$)C_6H_4**	**Fab25**	**R = 4-tBuC_6H_5**
**Fab08**	**R = 1,1`-biphenyl**	**Fab17**	**R = 2,4,6-$(CH_3)_3C_6H_2$**		

N-Triflylphosphoramides

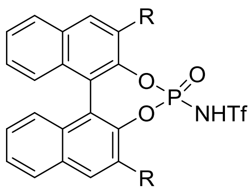

Fa (***R***) **Fb** (***S***)

**Fac01**	R = Ph	**Fac10**	R = 4-$CH_3OC_6H_4$	**Fac19**	R = 3,5-$(CH_3)_2C_6H_3$
**Fac02**	R = 9-phenanthryl	**Fac11**	R = 1-naphthyl	**Fac20**	R = 4-(4´-*t*$BuC_6H_4)C_6H_4$
**Fac03**	R = 9-anthracyl	**Fac12**	R = 2,4,6-(*i*$Pr)_3C_6H_2$	**Fac21**	R = 4-(4´-tBu-2´,6´-$(CH_3)_2C_6H_3)C_6H_4$
**Fac04**	R = 3,5-$CF_3C_6H_3$	**Fac13**	R = 4-Cl C_6H_4	**Fac22**	R = CF_3
**Fac05**	R = 4-$NO_2C_6H_4$	**Fac14**	R = $POPh_2$	**Fac23**	R = 3,5-(*t*$Bu)_2C_6H_3$
**Fac06**	R = 1,1`-biphenyl	**Fac15**	R = 4-$CH_3C_6H_4$	**Fac24**	R = $SiCH_3Ph_2$
**Fac07**	R = $SiPh_3$	**Fac16**	R = 3,5-F_2Ph	**Fac25**	R = 4-*t*BuC_6H_5
**Fac08**	R = H	**Fac17**	R = 4-(2´,4´,6´-$(CH_3)_3C_6H_2)C_6H_4$		
**Fac09**	R = 2-naphthyl	**Fac18**	R = 2,4,6-$(CH_3)_3C_6H_2$		

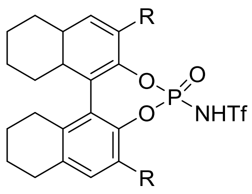

Fa (***R***) Fb (***S***)

**Fad01**	R = $SiPh_3$	**Fad10**	R = 2,4,6-(*i*$Pr)_3C_6H_2$	**Fad19**	R = 4-(4´-tBu-2´,6´-$(CH_3)_2C_6H_3)C_6H_4$
**Fad02**	R = 2-naphthyl	**Fad11**	R = 4-ClC_6H_4	**Fad20**	R = CF_3
**Fad03**	R = 9-phenanthryl	**Fad12**	R = $POPh_2$	**Fad21**	R = 3,5-(*t*$Bu)_2C_6H_3$
**Fad04**	R = 3,5-$(CF_3)_2C_6H_3$	**Fad13**	R = 4-$CH_3C_6H_4$	**Fad22**	R = $SiCH_3Ph_2$
**Fad05**	R = 4-$NO_2C_6H_4$	**Fad14**	R = 3,5-FC_6H_4	**Fad23**	R = Ph
**Fad06**	R = 1,1`-biphenyl	**Fad15**	R = 4-(2´,4´,6´-$(CH_3)_3C_6H_2)C_6H_4$	**Fad24**	R = 9-anthracenyl
**Fad07**	R = H	**Fad16**	R = 2,4,6-$(CH_3)_2C_6H_3$	**Fad25**	R = 4-*t*BuC_6H_5
**Fad08**	R = 4-$CH_3OC_6H_4$	**Fad17**	R = 3,5-$(CH_3)_2C_6H_3$		
**Fad09**	R = 1-naphthyl	**Fad18**	R = 4-(4´-*t*$BuC_6H_4)C_6H_4$		

Miscellaneous

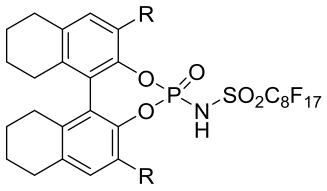

Fa (***R***) **Fb** (***S***)

**Faj01**	R = $SiPh_3$	**Faj10**	R = 2,4,6-(iPr)$_3C_6H_2$	**Faj19**	R = 4-(4´-tBu-2´,6´-(CH_3)$_2C_6H_3$)C_6H_4
**Faj02**	R = 2-naphthyl	**Faj11**	R = 4-ClC_6H_4	**Faj20**	R = CF_3
**Faj03**	R = 9-phenanthryl	**Faj12**	R = $POPh_2$	**Faj21**	R = 3,5-(tBu)$_2C_6H_3$
**Faj04**	R = 3,5-(CF_3)$_2C_6H_3$	**Faj13**	R = 4-$CH_3C_6H_4$	**Faj22**	R = $SiCH_3Ph_2$
**Faj05**	R = 4-$NO_2C_6H_4$	**Faj14**	R = 3,5-FPh	**Faj23**	R = Ph
**Faj06**	R = 1,1`-biphenyl	**Faj15**	R = 4-(2´,4´,6´-(CH_3)$_3C_6H_2$)C_6H_4	**Faj24**	R = 9- anthracyl
**Faj07**	R = H	**Faj16**	R = 2,4,6-(CH_3)$_2C_6H_3$	**Faj25**	R = 4-tBuC_6H_5
**Faj08**	R = 4-$CH_3OC_6H_4$	**Faj17**	R = 3,5-(CH_3)$_2C_6H_3$		
**Faj09**	R = 1-naphthyl	**Faj18**	R = 4-(4´-tBuC_6H_4)C_6H_4		

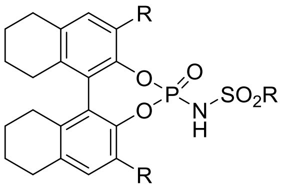

R = 4-$CF_3C_6H_4$
Fa (***R***) **Fb** (***S***)

**Fak01**	R = $SiPh_3$	**Fak10**	R = 2,4,6-(iPr)$_3C_6H_2$	**Fak19**	R = 4-(4´-tBu-2´,6´-(CH_3)$_2C_6H_3$)C_6H_4
**Fak02**	R = 2-naphthyl	**Fak11**	R = 4-ClC_6H_4	**Fak20**	R = CF_3
**Fak03**	R = 9-phenanthryl	**Fak12**	R = $POPh_2$	**Fak21**	R = 3,5-(tBu)$_2C_6H_3$
**Fak04**	R = 3,5-(CF_3)$_2C_6H_3$	**Fak13**	R = 4-$CH_3C_6H_4$	**Fak22**	R = $SiCH_3Ph_2$
**Fak05**	R = 4-$NO_2C_6H_4$	**Fak14**	R = 3,5-FPh	**Fak23**	R = Ph
**Fak06**	R = 1,1`-biphenyl	**Fak15**	R = 4-(2´,4´,6´-(CH_3)$_3C_6H_2$)C_6H_4	**Fak24**	R = 9- anthracyl
**Fak07**	R = H	**Fak16**	R = 2,4,6-(CH_3)$_2C_6H_3$	**Fak25**	R = 4-tBuC_6H_5
**Fak08**	R = 4-$CH_3OC_6H_4$	**Fak17**	R = 3,5-(CH_3)$_2C_6H_3$		
**Fak09**	R = 1-naphthyl	**Fak18**	R = 4-(4´-tBuC_6H_4)C_6H_4		

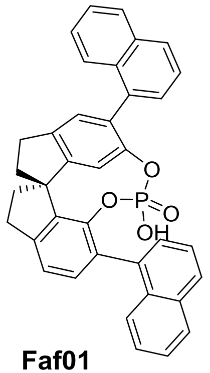

Faf01

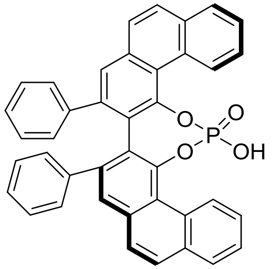

Fae01

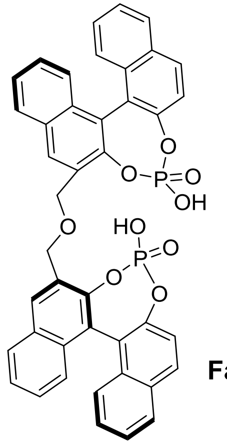

Fah01
